# Using high-throughput sequencing to explore the anti-inflammatory effects of α-mangostin

**DOI:** 10.1038/s41598-019-52036-5

**Published:** 2019-10-30

**Authors:** Peng Yin, Wenshu Zou, Jiandong Li, Na Jin, Qian Gao, Fenghua Liu

**Affiliations:** College of Animal Science and Technology, Beijing University of Agriculture (BUA), Beijing, P.R. China

**Keywords:** Inflammatory bowel disease, Inflammatory bowel disease, Inflammatory bowel disease, Gastroenterology, Gastroenterology

## Abstract

Lipopolysaccharide (LPS) causes an inflammatory response, and α-mangostin (α-MG) is an ingredient of a Chinese herbal medicine with anti-inflammatory effects. We investigated the mechanism by which α-MG reduces LPS-stimulated IEC-6 cells inflammation. A genome-wide examination of control, LPS-stimulated, and α-MG-pretreated cells was performed with the Illumina Hiseq sequencing platform, and gene expression was verified with quantitative real-time PCR (qPCR). Among the 37,199 genes profiled, 2014 genes were regulated in the LPS group, and 475 genes were regulated in the α-MG group. GO enrichment and KEGG pathway analyses of the differentially expressed genes (DEGs) showed that they were mainly related to inflammation and oxidative stress. Based on the transcriptomic results, we constructed a rat model of inflammatory bowel disease (IBD) with LPS and investigated the effects of α-MG on NLRP3 inflammasomes. After LPS stimulation, the rat intestinal villi were significantly detached, with congestion and hemorrhage; the intestinal epithelial cell nuclei were deformed; and the mitochondria were swollen. However, after pretreatment with α-MG, the intestinal villus congestion and hemorrhage were reduced, the epithelial nuclei were rounded, and the mitochondrial morphology was intact. qPCR and western blotting were used to detect NLRP3, caspase 1, interleukin (IL)-18, and IL-1β expression at the gene and protein levels. Their expression increased at both the transcript and protein levels after LPS stimulation, whereas it decreased after pretreatment with α-MG. This study provides new methods and ideas for the treatment of inflammation. α-MG may have utility as a drug for intestinal inflammation.

## Introduction

The small intestine is the largest digestive organ in the body. When inflammation occurs, the structure of the small intestine is damaged and a variety of inflammatory mediators, including proinflammatory and anti-inflammatory factors and chemokines, are released, which in turn damage the body^1–3^. IEC-6 cells are small intestinal epithelial cells that are important components of the mechanical barrier afforded by the small intestine, and their integrity plays an important role in intestinal immunity^4,5^. Because IEC-6 cells express Toll-like receptors (TLRs), including TLR2 and TLR4, they recognize LPS^6,7^, a component of the outer membranes of Gram-negative bacteria, and have been widely used for *in vivo* and *in vitro* studies of the inflammatory response^8–10^. Studies have shown that LPS increases the expression of inflammatory factors and chemokines, and causes physiological and morphological changes to tissues^11–14^. LPS regulates cytokine synthesis and release through different signaling pathways, including the nuclear factor κB (NF-κB), mitogen-activated protein kinase (MAPK), and Janus kinase–signal transducer and activator of transcription (JAK–STAT) pathways^15,16^. LPS also causes oxidative stress and increases reactive oxygen species (ROS) secretion^17,18^. NF-κB activation increases the secretion of NLRP3, whereas ROS promote the recruitment of apoptotic speck protein containing a caspase recruitment domain (ASC) and procaspase 1 by NLR family, pyrin domain containing 3 (NLRP3), leading to the activation of caspase 1, and activated caspase 1 cleaves pro-interleukin 18 (IL-18) and pro-IL-1β, and promotes the maturation of IL-18 and IL-1β^19–21^.

Traditional anti-inflammatory drugs, such as corticosteroid therapy, are widely used to relieve pain and inflammation, however, they interfere with the normal immune responses and may cause drug dependence^22–25^. Therefore, it is imperative to develop new anti-inflammatory drugs. Mangosteen peel is often used as a traditional medicine to treat wounds, wound infections, abdominal pain, and dysentery^26–28^. α-MG, the main component of mangosteen peel, is widely used for its anti-inflammatory and antioxidant properties^29,30^. It has been reported that α-MG exerts its anti-inflammatory effects by inhibiting the expression of tumor necrosis factor α (TNF-α), cyclooxygenase 2 (COX2), and prostaglandin-endoperoxide synthase 2 (PGE2), and inhibits the activation of the MAPK and NF-κB signaling pathways^6,31,32^. It has also been reported that α-MG exerts an antioxidant effect by inhibiting the production of ROS^26,33^. Although α-MG regulates the inflammation caused by a variety of factors, the underlying mechanism by which it regulates the LPS-induced inflammation of IEC-6 cells remains unclear.

Considering the important role of LPS in inflammation, the importance of IEC-6 cells in intestinal homeostasis, and the anti-inflammatory activity of α-MG, we used RNA-seq to perform a genome-wide exploration of the effects of α-MG on LPS-stimulated gene expression in IEC-6 cells, and verified the accuracy of the RNA-seq results using qPCR. To the best of our knowledge, this is the first study to use RNA-seq to assess the regulation of all gene expression in IEC-6 cells by α-MG. We show that α-MG effectively inhibited the LPS-induced inflammation of IEC-6 cells, and significantly downregulated the expression of inflammatory genes. We also constructed a rat model of enteritis using LPS and treated these rats with α-MG in *in vivo* experiments. Our results show that α-MG effectively protected the structure of the small intestine and inhibited the production of NLRP3 inflammasomes. Our data provide the first evidence that NLRP3 is critical to the molecular mechanism underlying the anti-inflammatory and protective effects of α-MG, which may be a novel drug for the treatment of enteritis and use in clinical research.

## Materials and Methods

### Ethic statement

All protocols involving animals were conducted in accordance with standards approved by Beijing Administration Office of Laboratory Animal (Approval Number: SYXK 2015-0004).

### Cell sample preparation

The IEC-6 cell line (CRL21592), purchased from the Cell Resource Center (Beijing, China), was grown in Dulbecco’s modified Eagle’s medium (Gibco, NY, USA) supplemented with 10% fetal bovine serum (FBS) (Gibco). The cells were grown in a 37 °C humidified incubator under 95% air and 5% CO_2_. *Escherichia coli* 055: B5 LPS (10 μg/mL; Sigma-Aldrich, MO, USA) was used to stimulate the cells for 12 h, before LPS processing, pretreatment with DMSO (concentration < 0.1%) for 1 h. α-MG [>98% high-performance liquid chromatography (HPLC) purity] were purchased from TongTian (Shanghai, China), α-MG are dissolved in DMOS to prepare a final concentration of 20 mM mother liquor for treatment of IEC-6 cells. The α-MG group was pretreated with 10 μM α-MG for 1 h and then stimulated with 10 μg/ml LPS for 12 h. TRIzol Reagent (Sigma, MO, USA) was added to the cells, which were stored at −80 °C. Total RNA was extracted from the cells, and the concentration and purity of the RNA were detected with a NanoDrop 2000 spectrophotometer (Thermo, MA, USA). The RNA integrity was confirmed with agarose gel electrophoresis. Quantitative analysis of RNA-seq was performed by Majorbio (Shanghai, China).

### cDNA preparation for microarray assay

The total amount of RNA used in building a database is 1 μg. mRNA was isolated from the total RNA using the A–T base pairing between oligo (dT)-conjugated magnetic beads and the 3′ ends of the eukaryotic transcripts. By adding the fragmentation buffer, the mRNA can be randomly broken into small fragments of about 300 bp. Six-base random primers (random hexamers) were added with reverse transcriptase, to synthesize the first-strand cDNA. Subsequent two-strand synthesis to form a stable double-stranded structure. The double-stranded DNA structure is a sticky end, which is added to the End Repair Nix to make up the blunt end, followed by an A base at the 3′ end for the y-shaped linker. PCR amplification for 15 cycles, 2% agarose gel recovery target band, TBS380 quantification, bridge PCR amplification on Cbot, followed by Illumina Hiseq sequencing. In this study, the transcriptome was sequenced with the Illumina Hiseq sequencing platform, and an Illumina paired-end (PE) library (300 bp) was constructed for 2 × 150 bp sequencing. The sequencing data obtained were quality-tested and analyzed with bioinformatics.

### Library data analysis

The raw data were first filtered, and the clean data thus obtained were compared with the reference genes of the species. The fragments per kilobase per million reads (FPKM) method was used to calculate gene expression, and the DEGs were identified with the DESeq2 software. The significance of the DEGs was determined with a KEGG enrichment analysis and a GO enrichment analysis, with the hypergeometric distribution test.

### qPCR

The total RNA was extracted from the IEC-6 cells and intestinal tissue using the Total RNA Kit I (Omega, CT, USA). Quantitative polymerase chain reaction (PCR) analysis was carried out using the DNA Engine Mx3000P® (Agilent, CA, USA) fluorescence detection system against a double-stranded DNA-specific fluorescent dye (Stratagene, CA, USA) according to optimized PCR protocols. β-Actin was used as the normalization control. The cycling conditions were: 95 °C for 3 min, followed by 40 cycles of 95 °C for 15 s, 60 °C for 30 s, and 72 °C for 60 s. The genes examined included *Nlrp3*, *interleukin 23 subunit alpha (Il23a)*, *C-C motif chemokine ligand 2 (Ccl2)*, *C-X-C motif chemokine ligand 1 (Cxcl1)*, *Cd69 molecule (Cd69)*, *prostaglandin-endoperoxide synthase 2 (Ptgs2)*, *Fraser extracellular matrix complex subunit 1 (Fras1)*, *potassium voltage-gated channel subfamily H member 2 (Kcnh2)*, *cadherin*, *EGF LAG seven-pass G-type receptor 2 (Celsr2)*, *fatty acid desaturase 2 (Fads2)*, *neuroblastoma 1*, *DAN family BMP antagonist (Nbl1)*, *tribbles pseudokinase 3 (Trib3)*, *activating transcription factor 6 (Atf6)*, *interleukin 9 receptor (Il9r)*, *signal-induced proliferation-associated 1 (Sipa1)*, *low density lipoprotein receptor (Ldlr)*, *Cxcl17*, *Caspase1*, *Il18*, and *Il1b*. Expression levels were determined using the relative threshold cycle (CT) method as described by the manufacturer (Stratagene). The gene-specific oligonucleotide primers used for qPCR are listed in Table 1.

**Table 1:** Gene-Specific Oligonucleotide Primers Used for qPCR.

Gene	Serial number	Primer sequence	Size of the products (bp)
β-actin	NM_031144.3	Forward 5′-CCACCATGTACCCAGGCATT-3′	253
		Reverse 5′-AGGGTGTAAAACGCAGCTCA-3′	
Nbl1	NM_031609.1	Forward 5′-CAAGCCTGCGGCAAGGAACC-3′	144
		Reverse 5′-TCCAGGAGGTTCTTCAGGTTCAGG-3′	
Fads2	NM_031344.2	Forward 5′-TCTCAGATCACCGAGGACTTCAGG-3′	153
		Reverse 5′-GCCATTGCCGAAGTACGAGAGG-3′	
Cxcl17	NM_001107491.1	Forward 5′-GTAGGAGGCTCCAGGAAGATGGC-3′	193
		Reverse 5′-TGGCAGGCTCTGGAGTGCTTG-3′	
Ldlr	NM_001013938.1	Forward 5′-GCTGGACCTGGACCGACCTC-3′	153
		Reverse 5′-GCCTAGTATGCGAGATGACGATGC-3′	
Atf6	NM_001107196.1	Forward 5′-GGCTTCCTCCAGTTGTTCTGTCTC-3′	120
		Reverse 5′-GCTTCTCTTCCTTCAGTGGCTCTG-3′	
Celsr2	NM_001191110.1	Forward 5′-CAGCCAGTGTCAGTGTAACCATCC-3′	84
		Reverse 5′-CGTTGAGCCGCACCGTGTAC-3′	
Ptgs2	NM_017232.3	Forward 5′-TTCCAGTATCAGAACCGCATTGCC-3′	143
		Reverse 5′-CCGTGTTCAAGGAGGATGGAGTTG-3′	
Ccl2	NM_031530.1	Forward 5′-GCAGGTCTCTGTCACGCTTCTG-3′	112
		Reverse5′-GAATGAGTAGCAGCAGGTGAGTGG-3′	
Il9r	NM_017021.1	Forward 5′-CAAGGACCGTATCGTTGGAGTGAC-3′	81
		Reverse 5′-AGTCTGGCCTCGTAGATGGTATCG-3′	
Il23a	NM_130410.2	Forward 5′-CCAGTGTGGTGATGGTTGTGATCC-3′	113
		Reverse 5′-AGATGTCCGAGTCCAGCAGGTG-3′	
Fras1	NM_001191595.1	Forward 5′-CACAAGGAGCCGAACTGACCAAG-3′	180
		Reverse 5′-AACACCGCACTGATGACTCGTAAC-3′	
Kcnh2	NM_053949.1	Forward 5′-GGTCATCTACACAGCCGTCTTCAC-3′	124
		Reverse 5′-GAGGAGGTCCACTACAGCCAGAG-3′	
Cxcl1	NM_030845.1	Forward 5′-CCGCTCGCTTCTCTGTGCAG-3′	155
		Reverse 5′-GTCCTGGCGGCATCACCTTC-3′	
Cd69	NM_134327.1	Forward 5′-CTTGTGCTGTGCTCGTAGTGGTC-3′	131
		Reverse 5′-GCAGGAAGCAGCATGGTGGTC-3′	
Sipa1	NM_001004089.1	Forward 5′-TGCTGCCTTACACGCCTAATAACC-3′	97
		Reverse 5′-GCTCCTGGAACACGATGGTCAC-3′	
IL-1β	NM_012762.2	Forward 5′-GCAGGCAGTATCACTCATTGT-3′	221
		Reverse 5′-GGCTTTTTTGTTGTTCATCTC-3′	
IL-18	NM_019165.1	Forward 5′-TGATATCGACCGAACAGCCAACG-3′	88
		Reverse 5′-GGTCACAGCCAGTCCTCTTACTTC-3′	
caspase 1	NM_012762.2	Forward 5′-ATGGCCGACAAGGTCCTGAGG-3′	185
		Reverse 5′-GTGACATGATCGCACAGGTCTCG-3′	
Nlrp3	NM_001191642.1	Forward 5′-GAGCTGGACCTCAGTGACAATGC-3′	146
		Reverse 5′-ACCAATGCGAGATCCTGACAACAC-3′	
Trib3	NM_144755.2	Forward 5′-CCACATCTCTGGCTGCTTCTGC-3′	122
		Reverse 5′- TGGTCCAGGCTCAGGCTCATC-3′	

Primers were designed from the published sequences in the GenBank database under the indicated accession numbers *F* forward primer, *R* reverse primer.

### Animal test

Eighteen male Sprague Dawley rats (from Vital River Laboratory, Animal Science and Technology Corporation, Beijing, China), weighing 200 ± 20 g, were adapted to 25 °C and 60% relative humidity (RH), and maintained under a 12:12 h light:dark cycle. Food and water were provided ad libitum for 3 days. On day 4, the rats were randomly divided into the control group, the LPS-stimulated group, and the α-MG-treated group, each consisting of six rats, and placed in plastic cages with cork carpets (400 × 300 × 180 mm^3^). Feed (200 g) and water (400 mL) were provided daily.

### Processing and sampling

The rats were housed under controlled conditions (25 °C, 60% RH) throughout the treatment period. The rats in the LPS-induced group were injected LPS (10 mg/kg) intraperitoneally on day 6. The rats in the α-MG-treated group were administered α-MG (50 mg/kg) intragastrically for 5 days at 08:00 every day and injected LPS (10 mg/kg) intraperitoneally on day 6. The rats were killed 24 h later. The jejunal intestine was removed and immediately flushed with saline to remove all intestinal contents. Each intestinal section was divided into five pieces: two block was fixed in 4% paraformaldehyde for histological analysis; two was stored at −80 °C; and one was fixed in 2.5% glutaraldehyde.

### Hematoxylin–eosin (H&E) staining

Rat jejunum group was fixed in 4% paraformaldehyde. (1) Dehydration: The jejunal tissue was taken to be 3 mm thick, dehydrated with a gradient alcohol, transparent, dipped in wax, embedded in a wax block, frozen for 20 min, sliced 5 μm, baked. (2) Embedding: Paraffin sections were placed in fresh xylene, soaked for 10 min, repeated 3 times; placed in absolute ethanol, soaked for 3 min, repeated 3 times; placed in 95% ethanol, soaked for 3 min, repeated 2 times; place in 75% ethanol, soak for 3 min, repeat 2 times; rinse with distilled water for 1 minute, put in PBS buffer. (3) After staining with H&E (Sigma, MO, USA), the stained sections were dehydrated with pure alcohol, and the sections were made transparent by xylene, and finally sealed with a neutral resin, and observation under a DP71 Olympus microscope (Tokyo, Japan).

### Transmission electron microscopy

The jejunal tissues fixed in 2.5% glutaraldehyde. (1) Dehydration: The jejunal tissue after pruning is carefully cleaned with tap water first. Ethanol gradient dehydration in a refrigerator at 4 °C, 50% ethanol, 70% ethanol, 90% ethanol, 90% ethanol: 90% acetone (1:1), 90% acetone gradient dehydration for 20 min, 100% acetone at room temperature for 20 min at room temperature, repeated three times. (2) Embedding: pure acetone + embedding solution (2:1) was incubated for 3 h at room temperature, pure acetone + embedding solution (1:2) was incubated overnight at room temperature, and the pure embedding solution was embedded at 37 °C for 2 h. (3) Curing: overnight in an oven at 37 °C, standing in an oven at 45 °C for 12 h, and standing in an oven at 60 °C for 24 h. (4) Ultrathin microtome sliced 50 nm, 3% uranyl acetate-lead citrate double staining, observed by transmission electron microscope.

### Immunohistochemistry of paraffin sections (IHC-P)

Paraffin sections were prepared as for H&E staining. Antigen retrieval by reference to the primary antibody instructions. The appropriate amount of endogenous peroxidase blocker was added to the sample, which was incubated at room temperature for 10 min, and then rinsed with phosphate-buffered saline (PBS). The primary antibody was dropped onto the specimen and incubated at 37 °C for 60 min. Goat Anti-Rabbit IgG (HRP) (Abcam, Germany) was added to the specimen and incubated at room temperature for 20 min. Each sample was hybridized with a specific antibody directed against NLRP3 (1:500 dilution), caspase 1 (1:50 dilution), IL-1β (1:100 dilution) (Abcam), or IL-18 (1:500 dilution) (Abnova, Taiwan, China). Sections were observed under a DP71 Olympus microscope (Tokyo, Japan) before stained with DAB (Beyotime Biotechnology, Haimen, China) and hematoxylin.

### Western blotting

The proteins were extracted from the rat jejunum with a total protein extraction kit (Kaiji, Wuhan, China) and quantified with a BCA protein assay kit (Pierce, Rockford, USA). The proteins were separated with SDS-PAGE, electrotransferred onto nitrocellulose membranes (Pierce), and then hybridized with a specific antibody directed against NLRP3 (1:500 dilution), caspase 1 (1:1000 dilution), IL-1β (1:1000 dilution) (Abcam), or IL-18 (1:1000 dilution) (Abnova). The protein bands were normalized to that of β-actin (Cell Signaling Technology, Danvers, USA) to correct for differences in loading.

### Statistical analysis

The RNA-seq data were analyzed statistically with DESeq. 2 and the other data with one-way analysis of variance (ANOVA) in SPSS for Windows (version 20.0; SPSS Inc., Chicago, IL, USA). The results are expressed as means ± standard errors of the means (SEM). Differences were considered significant at *P* < 0.05. Image densities (integrated optical density/area) of immunohistochemistry were obtained from three independent villi using ImageJ (National Institutes of Health, USA).

## Results

### DEGs after LPS stimulation with or without α-MG pretreatment

**Figure 1:**
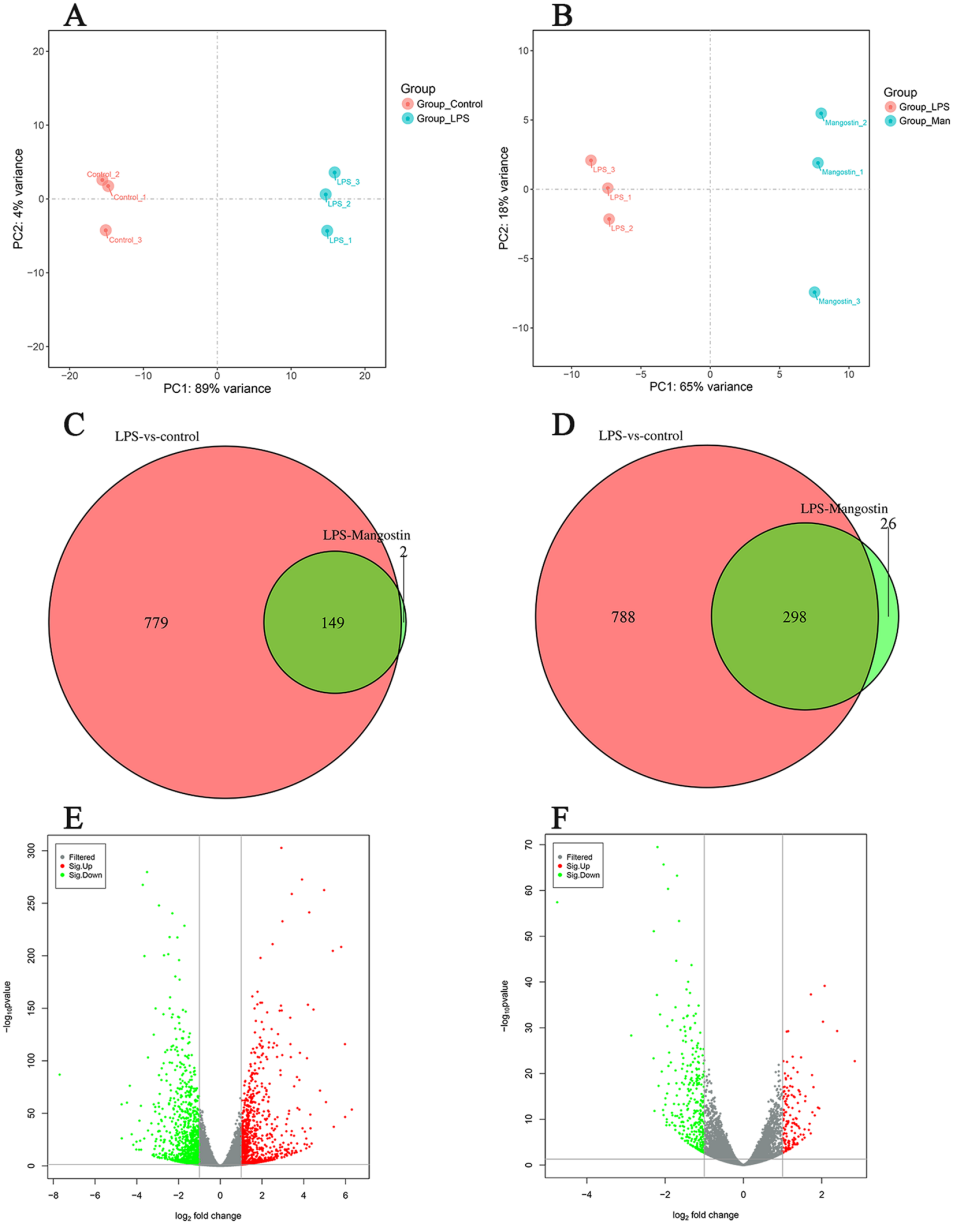
Strong changes in the transcriptome of IEC-6 cells stimulated with LPS or α-MG. (**A**) Principal components analysis (PCA) of RNA-seq data from IEC-6 cells in the LPS group (blue dots) and control group (red dots). (**B**) PCA of RNA-seq data from IEC-6 cells in the LPS group (red dots) and α-MG group (blue dots). (**C**) Venn diagram shows DEGs upregulated in the LPS group and α-MG group. (**D**) Venn diagram shows DEGs downregulated in the LPS group and α-MG group. (**E**) Volcano map showing the genes expressed in the LPS group, detected with RNA-seq. (**F**) Volcano map showing the genes expressed in the α-MG group, detected with RNA-seq. Colored dots indicate genes that do not differ significantly; red dots indicate genes with a log2 FC > 1 and *P* < 0.05; green dots indicate genes with log2 FC < −1 and *P* < 0.05. ‘Mangostin’ in the figure refers to α-mangostin.

**Figure 2:**
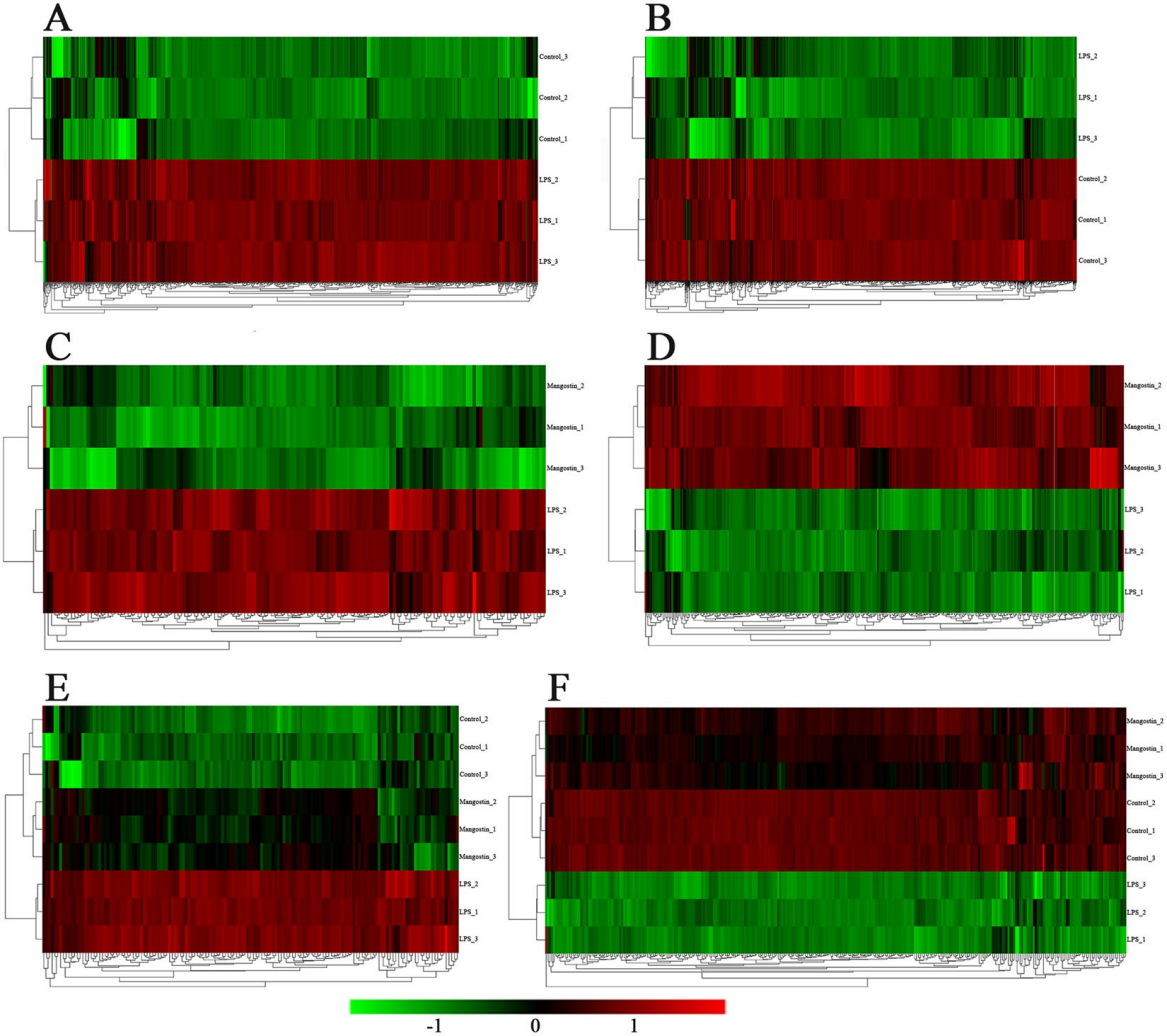
Hierarchical clustering and heat map of the RNA-seq data for DEGs in the LPS-stimulated or α-MG -pretreated IEC-6 cells. (**A**) In the LPS group, 928 DEGs were significantly upregulated compared with the control group. (**B**) In the LPS group, 1086 DEGs were significantly downregulated compared with the control group. The whole clusters are provided in Table S1. (**C**) In the LPS group, 151 DEGs were significantly upregulated compared with the α-MG group. (**D**) In the LPS group, 324 DEGs were significantly downregulated compared with the α-MG group. The whole clusters are provided in Table S2. (**E**) One hundred forty-nine DEGs were upregulated in both the LPS group and α-MG group. (**F**) Two hundred ninety-eight DEGs were downregulated in both the LPS group and α-MG group. The whole clusters are provided in Table S3. The screening criteria by which the DEGs shown in the figure were identified were P < 0.05 and log2 FC > 1. Red indicates upregulated genes and green indicates downregulated genes. ‘Mangostin’ in the figure refers to α-mangostin.

To assess the gene expression profiles in IEC-6 samples after LPS treatment with or without α-MG pretreatment, an RNA-seq analysis was performed using three independent biological replicates of each sample. We used FPKM as a measure of expression, and used an up- or downregulated log2 fold change (FC) > 1 or log2 fold change (FC) < −1 and *P* < 0.05 as the criteria with which to identify DEGs. When we subjected the gene expression levels to a principal components analysis (PCA). The PCA plot shows that the DEGs clusters in the control and LPS groups are good (Fig. 1A), good difference clustering between LPS group and α-MG group (Fig. 1B). By making the DEGs into a volcano map, it can be seen that there is a significant difference in gene expression after LPS treatment compared with the control group (Fig. 1E). Similarly, there are many DEGs in the LPS group compared with the α-MG group (Fig. 1F). A total of 32,883 genes were examined by combining the experimental data of the three groups. Among these, 928 genes were significantly upregulated (Fig. 2A) and 1086 significantly downregulated in the LPS group (Changes in gene expression in the LPS group relative to the control group) (Fig. 2B). These DEGs (2014 DEGs) are listed in Table S1, each small square above the heat map represents a DEGs, but because of its limited location, all genes are listed in the supplemental material. The highly expressed DEGs in the LPS group is shown in Table 2. In the α-MG group (Changes in gene expression in the LPS group relative to α-MG group), 151 genes were significantly upregulated (Fig. 2C) and 324 significantly downregulated relative to the LPS group (Fig. 2D). These DEGs (475 DEGs) are listed in Table S2, each small square of the heat map also represents a gene. The highly expressed DEGs in the α-MG group is shown in Table 3. After LPS stimulation, the numbers of upregulated and downregulated genes were similar, whereas after α-MG pretreatment, twice as many genes were downregulated than were upregulated. To further analyze the DEGs, Venn diagrams were used to describe the overlap of the upregulated genes. The coincidence of DEGs in the Control and α-MG groups was high. This indicates that the DEGs expression levels after LPS stimulation tended to be normalized by pretreatment with α-MG (Fig. 1C,D). The DEGs common to LPS and α-MG groups are listed in Table S3.

**Table 2:** LPS Group High upregulated Gene.

Gene ID	Gene Name	Log2FC	P-value
ENSRNOG00000056783	Cd69	6.529	<10^−5^
ENSRNOG00000007159	Ccl2	6.557	<10^−5^
ENSRNOG00000002802	Cxcl1	5.944	<10^−5^
ENSRNOG00000003254	Il23a	4.809	<10^−5^
ENSRNOG00000002525	Ptgs2	3.917	2.048E-273
ENSRNOG00000055156	Tnf	3.68	4.784E-22
ENSRNOG00000010278	Il6	2.576	0.00000022
ENSRNOG00000003170	Nlrp3	2.142	2.809E-45
ENSRNOG00000008622	Creb5	2.068	6.424E-11

**Table 3:** α-MG Group High upregulated Gene.

Gene ID	Gene Name	Log2FC	P-value
ENSRNOG00000056783	Cd69	6.529	<10^−5^
ENSRNOG00000003254	Il23a	4.809	<10^−5^
ENSRNOG00000002525	Ptgs2	3.917	2.048E-273
ENSRNOG00000020630	Il9r	3.677	2.206E-85
ENSRNOG00000010283	Cd28	3.161	9.891E-34
ENSRNOG00000020552	Fosl1	2.622	3.759E-45
ENSRNOG00000014835	Il1rl1	2.571	1.781E-08
ENSRNOG00000026607	Tnfsf18	2.481	1.309E-28
ENSRNOG00000003170	Nlrp3	2.142	2.809E-45
ENSRNOG00000008622	Creb5	2.068	6.424E-11

A large number of interleukins (*Il1a*, *Il23a*, *Il9r*, *Il24*, *Il33*, and *Il6r*) and chemokines (*Cxcl1*, *Cxcl11*, *Cxcl10*, *Cxcl2*, *Cxcl16*, *Cxcl17*, *Cxcl3*, *Ccl2*, *Ccl7*, *Ccl5*, and *Ccl20*) were included among the DEGs, in addition to the inflammation-related genes *Nlrp3* and *Ptgs2*.

### Gene Ontology (GO) analysis of DEG enrichment after LPS stimulation or α-MG pretreatment

**Figure 3:**
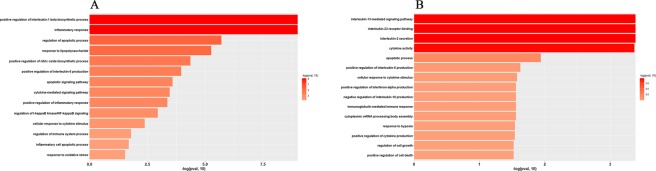
The GO database was used to classify the biological processes (BP) in which the DEGs after LPS stimulation or α-MG pretreatment were involved. (**A**) Fourteen pathways in which the DEGs in the LPS group were significantly enriched. The whole clusters are provided in Table S4. (**B**) Fifteen pathways in which the DEGs in the α-MG group were significantly enriched. The whole clusters are provided in Table S5. The Y-axis shows the relevant biological pathway, and the X-axis shows the score for each pathway (Fisher’s exact test was used to calculate the negative logarithmic enrichment for each pathway; enrichment was significant at *P* < 0.05). ‘Mangostin’ in the figure refers to α-mangostin.

To investigate the types of genes altered by LPS stimulation or α-MG pretreatment, the DEGs were subjected to a GO analysis. Genes can be classified into three levels: biological process (BP), molecular function (MF) or cellular component (CC), but we mainly focus on biological processes. Significant differences in the GO distributions of the DEGs in the different groups were defined as *P* < 0.05. The DEGs after LPS stimulation were mainly enriched in the GO terms: inflammatory response, regulation of apoptotic process, response to lipopolysaccharide, positive regulation of nitric oxide biosynthetic process, and positive regulation of IL-6 production (Fig. 3A). The DEGs associated with each GO term are shown in Table S4. The DEGs after α-MG pretreatment were mainly enriched in the GO terms: positive regulation of cell death, regulation of cell growth, positive regulation of cytokine production, response to hypoxia, and positive regulation of interferon-α production (Fig. 3B). The DEGs associated with each GO term are shown in Table S5. Thus, the GO analysis indicated that the two groups of DEGs (affected by either LPS or α-MG) were mainly associated with the immune response, and also closely associated with oxidative stress.

### Typical pathways regulating LPS stimulation and α-MG preconditioning

**Figure 4:**
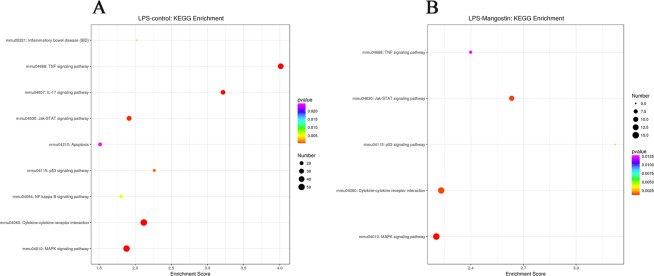
The KEGG database was used to analyze the DEGs in IEC-6 cells after LPS stimulation or α-MG pretreatment. (**A**) Nine pathways in which the DEGs in the LPS group were significantly enriched. The whole clusters are provided in Table S6. (**B**) Five pathways in which the DEGs in the α-MG group were significantly enriched. The whole clusters are provided in Table S7. The pathways are shown in a bubble diagram. The Y-axis shows the relevant pathways, the bubble size and color represent the score for each pathway (Fisher’s exact was used to calculate the negative logarithmic enrichment for each pathway; enrichment was significant at *P* < 0.05). The larger the bubble and the closer the color is to red, the stronger is the association. ‘Mangostin’ in the figure refers to α-mangostin.

To determine the relevant biological pathways involved in these effects, the Kyoto Encyclopedia of Genes and Genomes (KEGG) database was used to analyze the deep-sequencing data. Significant differences in the pathway distributions of the DEGs in the different groups were defined as *P* < 0.05. The pathways affected by the DEGs after LPS stimulation were IBD, the TNF signaling pathway, the NF-κB signaling pathway, the JAK–STAT signaling pathway, and other pathways (Fig. 4A). The DEGs associated with each pathway are shown in Table S6. The pathways affected by the DEGs after α-MG pretreatment were the TNF signaling pathway, the JAK–STAT signaling pathway, the TP53 signaling pathway, the cytokine-cytokine receptor interaction, and other pathways (Fig. 4B). It can be seen from the enriched signaling pathway that the differential genes are mainly related to inflammation. The DEGs associated with each pathway are shown in Table S7.

### qPCR validation of RNA-seq results

**Figure 5:**
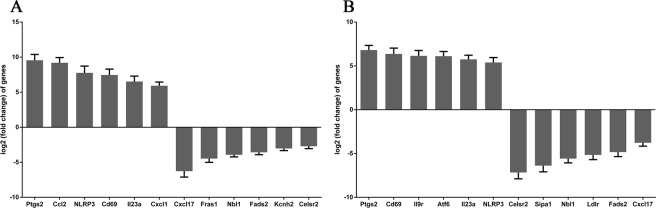
qPCR validated the IEC-6 RNA-seq results after LPS stimulation or α-MG pretreatment. (**A**) Six upregulated and six downregulated differentially expressed genes in the LPS group. (**B**) Six upregulated and six downregulated differentially expressed genes in the α-MG group. Data are the means ± SEM of three parallel experiments.

To validate the results of the RNA-seq analysis, six upregulated genes (*Nlrp3*, *Il23a*, *Ccl2*, *Cxcl1*, *Cd69*, and *Ptgs2*) and six downregulated genes (*Cxcl17*, *Fras1*, *Kcnh2*, *Celsr2*, *Fads2*, and *Nbl1*) in the LPS group were selected (Fig. 5A). Six upregulated genes (*Nlrp3*, *Ptgs2*, *Atf6*, *Il23a*, *Il9r*, and *Cd69*) and six downregulated genes (*Nbl1*, *Celsr2*, *Fads2*, *Cxcl17*, *Sipa*, and *Ldrl*) in the α-MG group were also selected (Fig. 5B). The relative expression levels of the test genes showed the same trends on qPCR as in the RNA-seq analysis, confirming the accuracy of the RNA-seq data.

### Effect of α-MG on jejunal tissues of rats with LPS injury

**Figure 6:**
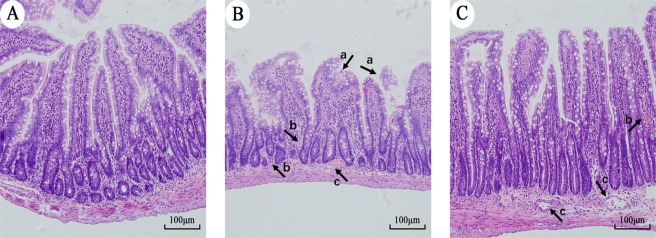
H&E staining was used to observe jejunal injury in rats. (**A**) Control group jejunum. (**B**) LPS group jejunum. (**C**) Jejunum of the α-MG group. (a) Exfoliation of the intestinal villi, (b) bleeding, and (c) hyperemia. Magnification, ×200.

Based on the RNA-seq results, we constructed a rat model of IBD using LPS and explored the anti-inflammatory effects of α-MG. H&E-stained sections showed that the normal rat jejunal villi were regularly arranged and compact, with an intact morphology and no congestion or bleeding (Fig. 6A). The LPS-stimulated rats showed obvious jejunal damage. The intestinal villi were loosely arranged, swollen, and shorter than normal, the tips of the villi were obviously detached, and the lamina propria was exposed, accompanied by obvious bleeding and congestion (Fig. 6B). After pretreatment with α-MG, the degree of swelling of the intestinal villi clearly improved to almost normal, the tips of the intestinal villi were relatively intact, the damage to the lamina propria was reduced, congestion was obviously improved, and bleeding was significantly reduced (Fig. 6C).

### Effects of α-MG on jejunal ultrastructure in rats with LPS injury

**Figure 7:**
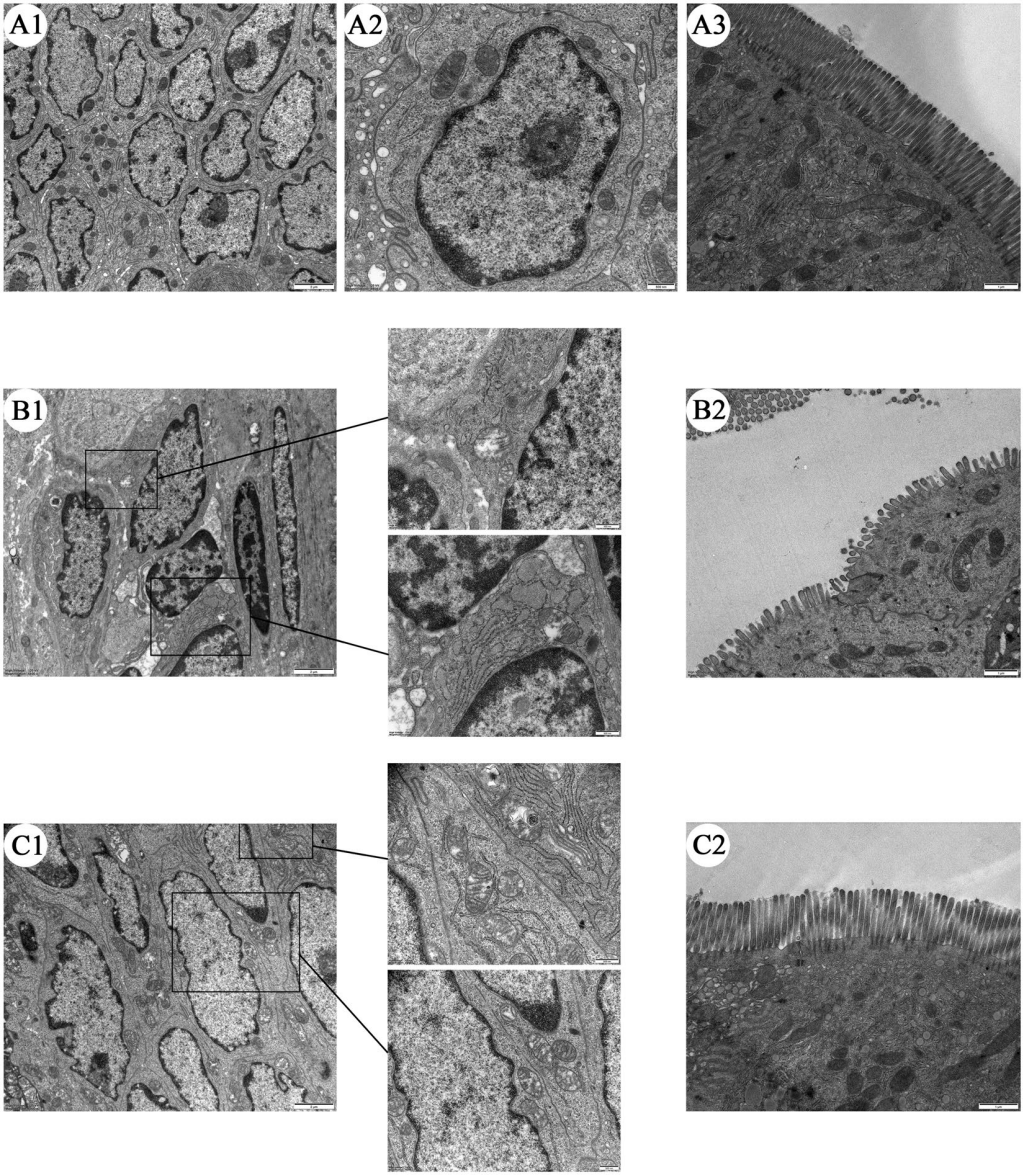
Transmission electron microscopic observation of the ultrastructure of rat jejunal tissues. (**A**) Control group jejunum, (A1) represents a 10,000-fold enlargement of the intestine, (A2) represents a 15,000-fold enlargement of the intestine, (A3) represents a 15,000-fold enlargement of the microvilli. (**B**) LPS group jejunum, (B1) represents a 10,000-fold enlargement of the intestine, (B2) represents a 15,000-fold enlargement of the microvilli. (**C**) Jejunum of the α-MG group, (C1) represents a 10,000-fold enlargement of the intestine, (C2) represents a 15,000-fold enlargement of the microvilli. “2 μm” indicates ×10,000 magnification, and “500 nm” indicates ×15,000 magnification.

To further investigate the damage to the rat jejunum, we observed the intestinal tract with transmission electron microscopy. The columnar epithelial cells in the control group are well formed and regularly arranged; the cell nuclei were round with a well-defined shape and some abnormal chromatin; and the mitochondria were intact, with no significant damage (Fig. 7A1,A2); microvilli are arranged tightly and without broken (Fig. 7A3). After the LPS treatment, the cells were disordered; the nuclei had shrunk and the abnormal chromatin in the nuclei had increased; there were early symptoms of necrosis and apoptosis; and the mitochondria were clearly swollen and the mitochondrial ridge was seriously damaged (Fig. 7B1); microvilli are loosely arranged, shortened and broken (Fig. 7B2). After pretreatment with α-MG, the cellular arrangement was significantly more regular than that in the LPS group; the nuclei were only slightly shrunken; mitochondrial swelling was significantly improved, and the mitochondrial ridge morphology was intact or only slightly fractured (Fig. 7C1); microvilli are arranged closely and there is almost no break (Fig. 7C2), however, the tissue was still slightly more damaged than the normal tissue.

### Effect of α-MG on LPS-induced inflammatory activation of NLRP3

**Figure 8:**
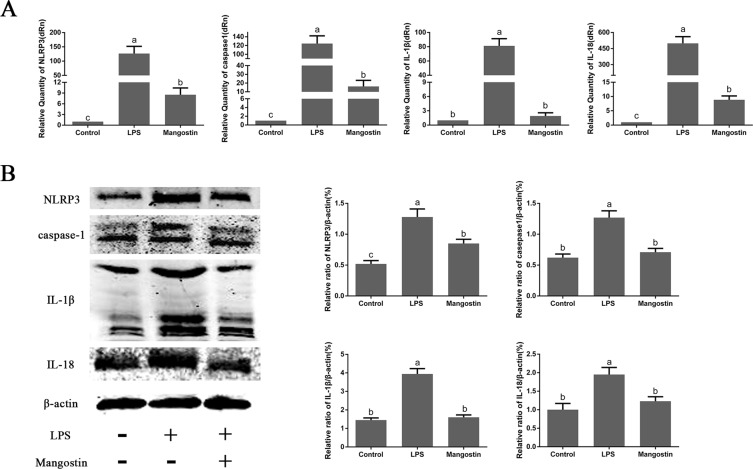
qPCR and western blotting were used to detect the expression of NLRP3, caspase 1, IL-1β, and IL-18 mRNAs and proteins. (**A**) qPCR was used to detect the expression of NLRP3, caspase 1, IL-1β, and IL-18 mRNAs in the rat jejunum. (**B**) Western blotting was used to detect the expression of NLRP3, caspase 1, IL-1β, and IL-18 proteins in the rat jejunum. Data are the means ± SEM of three parallel experiments. Different letters represent significant differences, that is the data between a and b, a and c, b and c shown in the figure indicate significant difference (*P* < 0.05). ‘Mangostin’ in the figure refers to α-mangostin.

**Figure 9:**
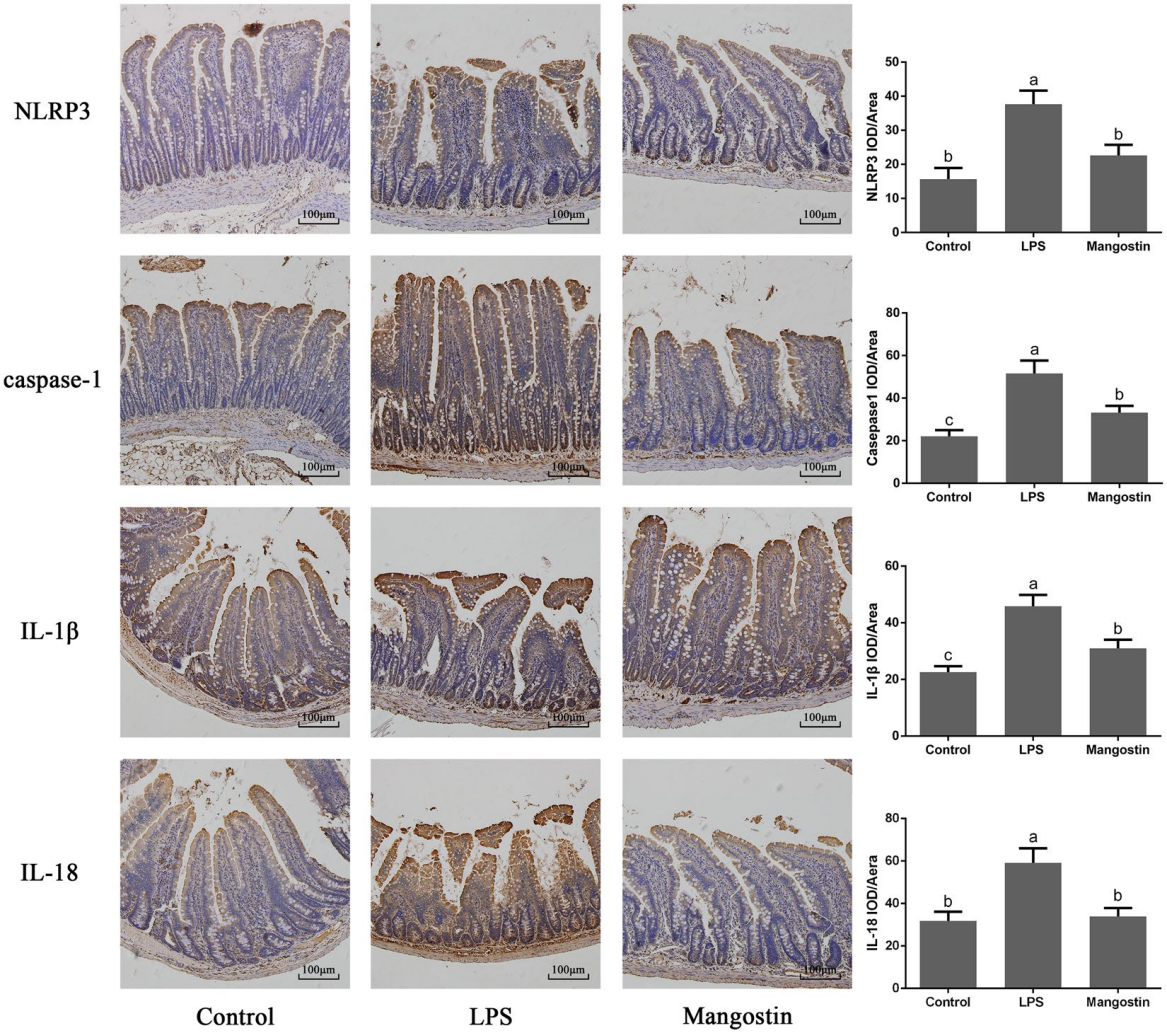
Expression of NLRP3, caspase 1, IL-1β, and IL-18 proteins in the rat jejunum was detected in paraffin sections with immunohistochemistry. Data are mean image densities (integrated optical density/area) ± SEM. The data is the result of processing the area of the three intestinal villi and calculating their average fluorescence density. Average fluorescence density calculated using Imagepro Plus software. Different letters represent significant differences, that is the data between a and b, a and c, b and c shown in the figure indicate significant difference (*P* < 0.05). ‘Mangostin’ in the figure refers to α-mangostin.

To verify the correlation between the anti-inflammatory effects of α-MG and NLRP3 inflammatory corpuscles, the expression levels of NLRP3, caspase 1, IL-1β, and IL-18 mRNAs were detected with qPCR. Their expression was significantly elevated after LPS stimulation, whereas their expression was significantly reduced after the administration of α-MG (Fig. 8A). We also determined the expression of NLRP3, caspase-1, IL-1β, and IL-18 with western blotting, and the their expression was significantly increased after LPS stimulation compared with that in the control group, but was significantly reduced after pretreatment with α-MG (Fig. 8B). The immunohistochemistry results also demonstrated that the expression of the NLRP3, caspase 1, IL-1β, and IL-18 proteins was significantly increased after LPS stimulation compared with that in the control group. However, their expression was also significantly reduced after the administration of α-MG (Fig. 9).

## Discussion

In this study, the transcriptome of IEC-6 cells changed after stimulation with LPS or pretreatment with α-MG. A total of 37,199 genes were expressed in IEC-6 cells. There were 2014 DEGs in the LPS group, 928 of which were upregulated and 1086 downregulated. There were 475 DEGs in the α-MG group, 324 of which were downregulated and 151 upregulated. We validated the RNA-seq results using the qPCR technology and demonstrated the reliability of the RNA-seq results. Our results identify, for the first time, the genes expressed in IEC-6 cells after treatment with LPS and all the genes through which α-MG exerts its anti-inflammatory effects. These results indicate that LPS stimulation and α-MG pretreatment induce significant changes in the expression of inflammation-related genes, such as those encoding interleukins, chemokines, NLRP3, and Ptgs2. Our GO enrichment and KEGG pathway analyses indicated that these DEGs are mainly associated with inflammation and oxidative stress.

Both interleukins and chemokines play important roles in the inflammatory response. Many members of the interleukin family act directly as inflammatory mediators to trigger inflammation^34,35^, whereas chemokines participate in inflammation through the chemotaxis of leukocytes^36,37^. Studies have shown that the elevated expression of IL-1β, IL-6, and IL-23 triggers inflammation^38–40^, whereas the expression of the anti-inflammatory factor IL-10 is reduced in the presence of inflammation^41,42^. IBD is a chronic inflammatory disease and is closely associated with damaged epithelial cells^38,43^. Our results show that after LPS stimulation, the ‘inflammatory bowel disease’ (IBD) pathway changed significantly. IL-23a was the major DEG in the IBD pathway. Recent studies of IL-23 have mainly focused on the regulation of IL-23a^44,45^. The stimulation of natural killer (NK) cells with LPS increases IL-23 expression, which acts together with IL-18. Our data demonstrated elevated IL-23 expression after LPS stimulation. However, the expression of IL-23a decreased after pretreatment with α-MG, and the GO pathway ‘negative regulation of interleukin-10 production’, in which it is involved, also changed significantly. IL-10 is an anti-inflammatory factor that inhibits the elevation of IL-1β and TNF-α induced by LPS^46,47^. It inhibited the expression of inflammatory cytokines IL-6 and TNF-α in LPS-stimulated primary mouse mixed glial cultures^46^. One of the CXC chemokines detected in this study, CXCL17, was discovered only 12 years ago and has been reported in very few papers. It has anti-inflammatory, antibacterial, and antiapoptotic effects, and elevated CXCL17 expression reduces the LPS-induced inflammatory response^48,49^. Our RNA-seq analysis showed that the expression levels of Cxcl17 and Il23a also decreased after LPS stimulation, but increased significantly after α-MG pretreatment. This implies that α-MG regulates the expression of interleukins and chemokines to exert its anti-inflammatory effect.

Oxidative stress is involved in the progression of the inflammatory response, and LPS causes oxidative stress and increases ROS production, leading to inflammation^50,51^. The hearts of rats exposed to LPS showed elevated levels of lipid peroxidation products and slightly elevated nicotinamide adenine dinucleotide phosphate (NADPH) oxidase activity^52^. The intraperitoneal injection of mice with LPS caused the activation of the TLR4 signaling pathway in their kidney tissues, causing oxidative stress^51^. The stimulation of Raw264.7 macrophages with LPS increased ROS production, which exacerbated inflammation^53^. Our results show that the GO ‘response to oxidative stress’ pathway was altered in LPS-stimulated IEC-6 cells, which also demonstrates that LPS causes oxidative stress and increases ROS production in IEC-6 cells. Ptgs2, also known as COX2, is encoded by a DGE in the ‘response to oxidative stress’ pathway and is a key rate-limiting enzyme in the synthesis of the inflammatory mediator PGE2^54,55^. The expression of Ptgs2 is tightly regulated by signals from molecular and pathway networks, including ROS, MAPK, and NF-κB, and its expression is elevated in the LPS-induced inflammatory response^56,57^. The stimulation of Raw264.7 macrophages with LPS caused an increase in PGE2 and COX2 expression and was associated with the activation of NF-κB^56,58^. LPS stimulates ROS generation and COX2 expression in adrenocortical cells, which are caused by the activation of MAPK^59^. Our data also show that LPS stimulation increased Ptgs2 expression. NF-κB, a classical molecule of the inflammatory response, degrades IκB after LPS stimulation, activates NF-κB, and induces the increased transcription of downstream inflammatory factors TNF-α and IL-6^60,61^. TNF is a proinflammatory factor that is elevated in the LPS-induced inflammatory response, and LPS-induced bone-marrow-derived macrophages cause elevated TNF-α expression^62–65^. In this study, Ptgs2 expression decreased after pretreatment with α-MG, and the TNF signaling pathway was significantly altered, which indicates that LPS stimulation causes NF-κB, activation, leading to increased Ptgs2 expression. However, α-MG exerted an anti-inflammatory effect by regulating the expression of Ptgs2. There was also a significant difference in the GO ‘regulation of IκB kinase/NF-κB’ signaling pathway after LPS stimulation, and NLRP3, a key molecule in the natural immune system, is encoded by a DEG in this pathway. The overactivation of NLRP3 is closely associated with inflammatory diseases^66,67^. NLRP3 inflammasomes affect inflammation by processing and activating proinflammatory cytokines, including IL-1β and IL-18, and the activation of caspase 1 is required during this process^68,69^. Therefore, the expression of the Nlrp3 gene was increased in NLRP3 inflammasomes after LPS stimulation, whereas its expression was reduced by pretreatment with α-MG. Therefore, we hypothesize that α-MG plays a key role in the anti-inflammation process by inhibiting the activation of NLRP3 inflammasomes.

Together, our RNA-seq data demonstrate that the expression of inflammation-related genes, such as Ptgs2, Nlrp3, and Il23a, is elevated after LPS stimulation, and that the expression of anti-inflammatory genes, such as Cxcl17, is reduced. The expression of inflammation-related genes decreased after α-MG stimulation, and the expression of anti-inflammatory genes was elevated, thus demonstrating that α-MG specifically regulates the expression of several inflammatory genes to exert its anti-inflammatory effects.

Based on the results of our RNA-seq analysis and the role of NLRP3 inflammasomes in inflammatory diseases, we used LPS to construct a model of IBD in rats and studied NLPR3 inflammasomes in this model, activated caspase 1 acts as a proinflammatory agent of cell death and is a key molecule in NLPR3 inflammasomes, which cause inflammation^70,71^. IL-18 and IL-1β are thought to be switches for the inflammatory response and play leading roles in the pathogenesis of IBD^72–74^. The NLRP3 inflammasomes are activated and the expression of caspase 1, IL-18, and IL-1β are increased in IBD^75,76^. There is evidence that NLRP3 inhibitors can be used as drugs for the treatment of IBD, thereby avoiding the adverse effects of corticosteroid therapies^77^. NLRP3-deficient mice with *Citrobacter rodentium*-induced IBD displayed more-severe intestinal inflammation and greater expression of ASC in their IECs than in infected NLRP3-sufficient mice, indicating that IECs are the major cell type involved in the activation of NLRP3^75^. Although this result is contrary to those of previous studies, it illustrates the importance of NLRP3 activation in IBD. Our results show that LPS increases the expression of NLRP3 and caspase 1 at both the mRNA and protein levels in the rat jejunum, and that the expression of the inflammatory factors IL-18 and 1L-1β at the mRNA and protein levels is also increased, which is consistent with the previous results. Although many studies have reported the anti-inflammatory properties of α-MG, no previous report has described the action of α-MG on NLRP3 inflammasomes. Our results demonstrate that the mRNA and protein levels of NLRP3, caspase-1, IL-1β, and IL-18 were reduced in LPS-treated rats pretreated with α-MG relative to those in rats treated with LPS only. This confirms that α-MG exerts its anti-inflammatory action by inhibiting the production of NLRP3 inflammasomes.

The intestinal integrity is the first barrier against environmental damage to the intestine. When bacterial inflammation occurs in the intestine, the intestinal tract is damaged, allowing bacteria to enter the bloodstream, with even more serious consequences^78–80^. Studies have shown that the inflammation of the intestine is mainly associated with damage to the intestinal villi, which predominantly manifests as their swelling and bleeding^81^. In LPS-induced enteritis, significant intestinal epithelial cells are shed and the intestinal villus height and crypt depth are reduced^82,83^. Our results show that after LPS stimulation, intestinal villus detachment was accompanied by significant hyperemia and hemorrhage, which was significantly improved after pretreatment with α-MG. Other studies have found that the inflammation of the intestine is closely related to the damage of the intestinal microvilli, and that the intestinal microvilli of rats with enteritis are shortened and loosely arranged^84,85^. In the present study, further observation with transmission electron microscopy revealed that the intestinal microvilli were sparsely arranged and shortened after LPS stimulation. It is noteworthy that after α-MG pretreatment, the morphology of the intestinal microvilli was almost the same as that of the normal intestinal microvilli. This indicates that α-MG prevents the intestinal damage caused by LPS, especially the damage to the intestinal microvilli.

In summary, in this study, we focused on the anti-inflammatory potential of α-MG. Our RNA-seq data demonstrate, for the first time, the changes in gene expression induced by α-MG in an IEC-6 cells model of inflammation, demonstrating that α-MG selectively inhibits the expression of several inflammation-related genes. Our animal experiments show that α-MG has a specific inhibitory effect on the production of NLRP3 inflammasomes, and may therefore be a candidate agent for the prevention of inflammatory diseases.

## Conclusions

This study is the first to discover that α-MG can inhibit the production of NLRP3 inflammasomes and thus exert anti-inflammatory effects, which provides a basis for the development of new anti-inflammatory drugs.

## Supplementary information

Dataset 1

## Data Availability

All data generated or analysed during this study are included in this published article (and its Supplementary Information files).
