# Supplementary material for: Using high-throughput sequencing to explore the anti-inflammatory effects of α-mangostin
Source: Sci Rep. 2019 Oct 30;9:15626. doi: 10.1038/s41598-019-52036-5 (PMC6821923; doi:10.1038/s41598-019-52036-5)
Supplement: Supplementary file 1 — Dataset 1 [file 41598_2019_52036_MOESM1_ESM.pdf]

Using high-throughput sequencing to explore the anti-inflammatory effects of α-mangostin

Peng Yin, Wenshu Zou, Jiandong Li, Na Jin, Qian Gao, Fenghua Liu

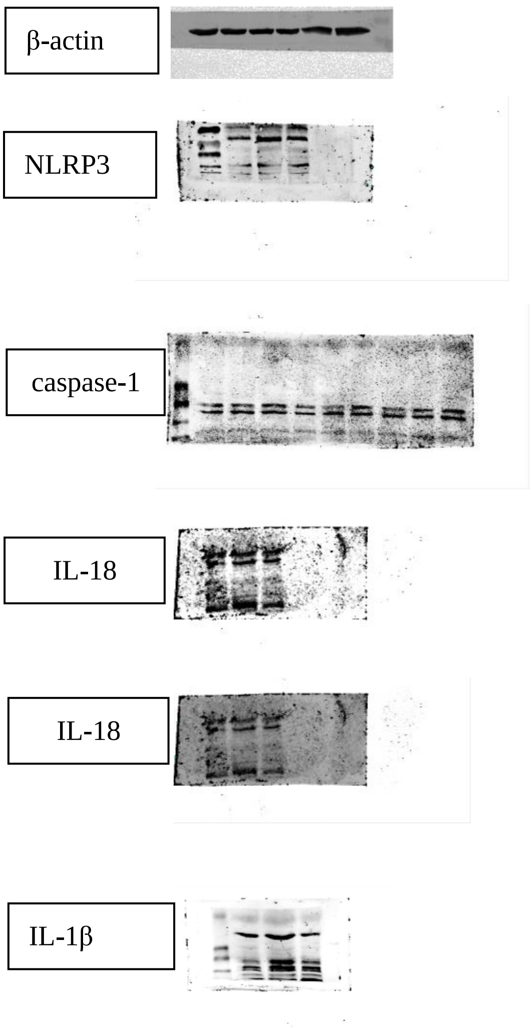

Fig. 8 qPCR and western blotting were used to detect the expression of NLRP3, caspase 1, IL-1β, and IL-18 mRNAs and proteins. (β-actin) The first lane to the third lane are the target strips of the β-actin of this experiment. (caspase-1) The fifth lane to the seventh lane are the target strips of this experiment caspase-1.

Table. S1: Lists of genes affected by LPS group

Transcriptome results	Genes	Total number
Up-regulated genes	AABR07054490.1, Fv1, Noc4l, Thpo, Creb5, Bdkrb1, Mapk11, Sox9, Popdc3, AABR07070238.3, Nhej1, Entpd1, Nfyb, Bmp6, Slc6a5, Gpr37l1, Nppb,Plekha4, St3gal6, Hspa5, Jph3, Lcn2, Zc3h8, Rhbdf2, Atp11a, Csf3, Gipc2, Tp53rk, Vegfa, Hes3, Pdzk1ip1,	928

	AABR07026032.3, Acsm1, Slc25a24, Gprc5d, Dnmt3b, Htatip2, Kbtbd8, Nhs,Nol10, Ikbke, Rmdn3, Mpa2l, Bid, Chchd6, Rdh10, Npm3, Micall2, Fgf21, Tubb3, Ern1, A1cf, Ptch2, Traf3, Traf2, Cplx2, Utp14a, Megf10, Fkbpl, Tsen2, Eprs, Faslg, Rpf2, Pgs1, Mx1, LOC100912485, Desi1, Rnd1, Rgs9, Irx2, Acsl1, Bdkrb2, Flrt2, Slc41a2, Nlrp6, Cdc42se2, ST7, Dntt, Cecr2, Ifngr2, RGD1561277, Timm9, Tnfaip2, Ripk2, Slc24a1, Rn60_20_0031.1, Plcl1, Myh3, Il17ra, Dnase2b, Sacm1l, Nop2, Tlr2, Pus1, MGC105649, Slc16a6, Tars, Muc13, Csf2, Sdhaf1, Slc23a2, Cers6, Timm10, Kcnh6, Foxn1, LOC108348080, Ctns, Yae1d1, Bop1, Otop1, Izumo1, Col6a5, Gbp4, Slc30a1, Mthfd2, Mthfd1l, Pdzd2, Islr2, Cyp4f39, Tgfa, Nt5dc3, Tnfrsf26, Pld5, Mrps18b, Sat1, Slc25a37, RT1-CE10, Thg1l, Mpp6, Parp8, Nlrp3, Il23a, Ank2, Slc35g1, Lyl1 ,Nr4a3, Pdzd3, Mir221, Nfkb2, Mnda, Il18rap, Exoc3l4, Cx3cl1, Rn50_X_0640.3, Nkain1, Myc, Kcnj1, Arntl, LOC299312, Eaf1, March3, Plekhg1, Fbrsl1, Fam49a, Akr1b10, Qtrt2, Cldn17, Wdr25, Tspan2, Tmem62, Mast2, Rrp8, Syne1, Ptgr2, Il9r, Rap1gap2 ,Wdr46, Scarf1, Dnajc25, Asb5, Sssca1, Krt17, Nrf1, Piezo2, Plxdc1, Cxcl11, St6galnac1, Rassf6, Arrdc4, Cfap100, Klb, Rundc3b, Cxcl10, Trmt6, Steap1, Gpr155, AABR07013410.4, Ecscr, Dgat2, Cdr2, Ccl2, Efr3b, Jaml, Hmox1, Zfp639, Rcn1, Oit3, Cd22, LOC497963, AABR07015002.1, Pmm2, Cmbl, Hk2, Cacna1s, LOC100910575, AC128394.1, Csf1, Nfxl1, S100a7a, S100a5, Ppp1r15b, Per2, Larp1, SNORA73, Siah2, SNORD93, Iars, Chadl, Slc24a2, AC111319.2, Vps37c, Nkrfl, Nox1, Nfkbia, Tfe3, Mrpl38, Etv4, Ccdc137, AABR07015674.1, Msh5, Neb, Eif1a, Chac1, Dgkk, LOC688459, Atp8b4, Cebpb, Ulk3, Ifit2, Pycr1, Sdad1, Klra2, Mmp9, Bcl3, Cfap58l1, Scn11a, Pigy, Abcc8, Peg12, Rab9b, Fermt1, Fut1, Psmb8, Optn, AABR07016976.1, Vgf, RT1-Da, SNORA9, Abca14, Slc26a9, Mmachc, Aqp8, Cpeb2, Ifi27l2b, Gfod1, MGC116197, Pappa, Hoxb9, LOC103691744, Slc5a11, Dph5, Erlin1, Cxcl3, Rapgef5, Ppm1h, Pkia, Cacna1d, Fam169a, LOC108348111, Bend3, AABR07067469.1, AABR07000159.4, Emp2, Zfp238, Il1a, Wars, Rdh13, Tnf, Phpt1, RGD1563354, Rpia, Mybbp1a, Ggt1, Sele, Ppp4r4, Rps6ka2, Cd300lf, Bcat1, Rn50_7_1163.2, Odc1, Map3k6, Serpinb2, Aars, Rasal3, LOC100912293, Nle1, Timm8a1, Fam57b, Clec4e, Crmp1, Cxcl2, AABR07026032.1, Tfrc, Usp10, Polr1e, Pak1ip1, SNORA32, Uprt, Letm1, Chi3l1, Col4a3, Tppp, Cd44, Slc16a7, Ddit3, Tspan33, Gpr63, Ckmt1, Robo2, Socs2, Postn, Atf5, Rwdd1, Cad, Lars, Slc7a5, SNORD12, Avil, Casp12, Baz1a, Fhad1, RGD1565367, NEWGENE_1306399, Rab15, Taf1a, Aen, Fam129a, Cabp1, Slc7a3, Scube3, Mt2A, Clec10a, Lrrc59, Nod2, Icam1, Trim66, Fas, SNORD49, Slc11a2, AABR07044473.1, Myt1, AC097183.1,	

	Arhgap4, SNORD22, Egr2, AABR07037356.1, Rbp2, Cd74, Nfil3, LOC103689945, Rhbdd1, Plekhs1, LOC100911575, Tslp, Pparg, Stard9, AABR07000629.2, Mcoln3, Abcg1, Plet1, Kdm6b, Srm, Mettl13, LOC103694381, Phlda1, Pcdh12, Stx6, Map1b, AABR07027096.1, Tap1, Fam171a1, Gcsh, Ifrd2, Tmem154, Manba, Sulf1, LOC100911453, Slc1a4, Relt, Map3k8, Atf3, Rn60_10_0648.2, Gbp2, Cdyl2, Pwp2, LOC100360856, Guca1b, Adam23, Plekha7, Denr, Pramef27, Slc4a7, Fads3, Mt1, AABR07058805.1, Tnfsf8, Akap2, Gar1, Srrd, Elovl2, Ttc39b, RGD1563365, Cfap45, Nrip2, Cyp19a1, Rrp12, Slc3a2, Vil1, Mab21l3, Mpv17l, Adgre1, Bicdl2, Tnfrsf9, AABR07047844.1, Uap1, Cacna1h, Plcxd2, AABR07014239.1, Pus7l, Grm1, Hivep1, Fa2h, Nol12, C1qbp, Aoc3, Ltv1, Gys1, Rn60_20_0054.5, Them4, Hap1, Ffar2, Pgam5, Fcamr, Mrm3, Emg1, AABR07050283.2, Elf3, Polr1b, Riok3, Tmprss7, Ctu2, Akna, Antxr2, Pim3, Galnt15, AABR07008256.1, Gpr68, Pik3r6, Wdr77, Nsun5, Zmynd19, Ercc1, Krt24, Stc1, Tnfaip6, Hoxc6, Klf4, Nuggc, Slc25a5, RT1-A2, Otub2, Cmah, Il24, Cmpk2, Rn60_2_1847.3, Tnfsf18, Klf6, Cflar, C3, Icoslg, Ghitm, Mat1a, Cfap43, Angptl4, Sfxn1, Stx11, Zeb2, Fsd1, Cox6a2, Spata5, Gcgr, snopsi28S-3327, Camk1g, Acot2, Coq10b, Nutm2f, Slco5a1, Ntsr1, NEWGENE_1306714, Ptpro, Sars, Il17f, Tnfsf15, Gch1, Tbl2, Vsig8, Stk32a, Rpl13a, Ehf, Atf4, Pla2g2a, Ptgs2, Esrra, Adpgk, Lyrm1, Il1rl1, Madcam1, Nfkbiz, Utp18, Fyn, Rn60_17_0530.1, Mars, Fkbp5, Casp4, Trem3, LOC100911572, Wdr12, Eef1e1, RT1-CE4, Actl7b, Arid5a, Foxred2, Serp1, Col7a1, Heatr1, Tnip3, Aloxe3, Vldlr, Abcf2, Etnppl, Dock6, RGD1307704, LOC100911440, Cars, Dll4, Lhfpl2, Manea, Donson, Heatr3, Ciapin1, Olr1, Gars, Mdn1, Cyp27b1, Inpp4b, Vat1l, Stbd1, Dcx, Creb3l1, Alg12, Kazn, Slc6a9, Aatf, RT1-CE1, Gtpbp2, RT1-CE7, Cebpg, Car12, Kif1a, Eif4ebp1, Etv5, Pprc1, Ccl20, Cd69, AABR07032393.1, Shmt2, Nqo2, Herpud1, Sox11, Lonrf1, Unc5c, Gna14, Alg1, Pinx1, Clca2, Foxh1, Chchd4, Neto2, LOC102551435, Ddx21, Plk2, Tma16, Tomm40, AC128848.1, LOC100912565, Adm2, Kctd15, Cdpf1, Ccl7, Bysl, Gramd1b, LOC103692471, Sh2d7, Ubash3b, Gadd45a, Glce, Lef1, Neurl3, Slpi, Dnah1, Irf5, Wdr43, Cd160, Mmp13, Tmem190, Chd7, Elmo1, Slc20a1, Angpt1, Cycs, Elavl2, Fjx1, P2ry2, Mrto4, SNORD50, AABR07060519.1, Ier2, Dlx5, Tnfsf13, Il36a, Nars, Serpinb3, Pax9, Gys2, Nlrx1, Fam185a, Ifrd1, Cnnm4, Grk2, Cacna1i, Rn50_10_0892.1, Senp5, Jmjd6, AABR07005779.4, Eif2s2, Uchl3, Slc25a33, Nfkbie, Eif2b3, Dus4l, Ly6e, Syt4, Mtr, Alkbh3, Psat1, Hspa9, Selp, Sod2, Jak2, Tigar, Wbscr22, Adam12, Ubd, Kctd19, Tmem132b, Nfkbib, Afdn, Dennd4a, Ehd3, Ccl5, Tnfaip3, Zmynd15, Phf10, Rn60_20_0141.4, Vars, RT1-CE16, Has2, Gpr3, Zc3h12a, Ruvbl2, Fosl1, Rcl1,	

	Tmem268, Taf4b, Inhbe, LOC100302465, Mettl2b, Lrrc8b, Fbn1, Wdr3, AABR07027272.1, Egr1, Selenos, Ddn, Bcan, Tmem158, NEWGENE_1309258, Nrcam, Ada, Prr5l, Dnajc21, Cxcl16, Fam167a, Hs3st5, Pck2, Mtfr1, P2ry6, Upp1, PVR, Myo5b, Pik3r5, RGD1562200, Irf8, Mill1, Fhl2, Lyrm9, Rbpj, Rsl24d1, Ptprz1, Naa25, Cry1, Abcc9, AABR07066820.1, LOC100910854, Atp13a4, LOC102546376, Mrps10, LOC100363502, Nolc1, Psmb10, Zfp280b, Ndufaf4, Cd28, RT1-Ba, Trib3, Il6st, Lama3, Card11, Dnajb9, Mdfic, Ppp1r15a, Slfn4, Tsnax, Noct, Mef2b, Anks6, Lmbr1, Epha2, Xpot, Aox2, Kif19, Ttyh2, Atp2a3, LOC103692087, Mycl, Ciart, Alg3, LOC100910732, Pmaip1, Sdcbp2, Hspa4l, snoR38, Chka, SNORD87, Fam71f1, Il6r, Lonp1, Cxcl1, Nmi, Grhl1, Cyp26b1, Prmt3, RT1-CE3, Usp36, Tapbp, Cd40, Psph, Ltb, Srf, Tp53, Eif1ad, Il6, Shc4, Gtpbp4, Adamts17, Bcs1l, Foxk2, Fcer2, Nkrf, LOC108351703, Dusp8, Rgn, Arg2, RT1-Db1, Cyr61, Fez1, Prr7, Fibin, Smyd5, Yars, Vcam1, Dusp4, Slc7a11, Mrm1, Kif21b, LOC108348062, Tap2, Ogfod1, Znf354b, Tenm2, Slc6a4, Cenpn, Slco3a1, Srgap1, Wdr74, Adora2b, Nop56, Abcb1a, Apln, Plcd4, Prss22, LOC100361898, Sytl2, AABR07050530.1, Golga7b, Acod1, Ptpn18, Ddr2, Raph1, Ccdc97, Thrb, Maff, Nid1, Aldh18a1, Sesn2, Rnf219, Tll1, Rab32, Phgdh, Pabpc6, Dhrs9, Gbp5, Igdcc4, Lif, LOC103692719, Asns, AABR07017110.1, Atmin, Wdyhv1, LOC499229, Hydin, Rbpms2, C1s, Mir155, Gpatch4, Lyrm4, Mknk1, Relb, Adgrg6, Ciita, AC109427.1, Calcrl, Hephl1, Ccdc86, Siae, Stap2, Cyp2u1, Bmper, Pglyrp3, Arhgef6, Rrs1, LOC500877, Mitf, LOC100912578, Farsb, Lysmd2, Birc3, RT1-Bb, Ptges2, Spata2, Ercc8, Slc16a14, Chic2, Atf6, AABR07017902.1, AC117058.1, Trpc6, Wdr4, Atmin, Fsip1, Traf1, Pdss1, Reln, Lefty1, Nnmt, Ier3, Dapk1, Ccnd2, Vnn3, Desi2, Kcnj3, Prdx3, Tmx4, Slc1a5, Rrp15, Slc35f2, AABR07025051.3, Hcar2, Slc26a8, Tmprss4, Rrp9, Slc7a1, Aldh1l2, AABR07054460.4, Frmd5, Fgfr2, Sema4a, Muc15	
Down-regulated genes	Pmp22, Gpsm1, Ypel4, Elfn2, Lrr1, Ugt1a5, Cd9, Uaca, Flvcr2, Zfp395, Tmem140, Kank4, Aldh2, Lbh, LOC100360908, Nod1, Mmp28, Mmd, Tmem119, Ttk, LOC108348215, Lrtm2, Mybphl, Csgalnact1, Ldhc, Tmem98, Ptk2b, Map1a, Mboat1, Rasl10b, Tdgf1, Phykpl, Cdh13, Sema3f, Aldh6a1, Nudt12, Pbk, Gng8, Alpk3, Prss30, Lss, Rn50_16_0006.1, Il20rb, Gabre, Fancd2, Abca8, Acot11, RGD1309350, LOC100910404, Arhgap27, Fam81b, Ogn, Ovol1, Micalcl, Hrct1, Nxph3, AABR07019061.1, Ybx2, Irf9, Acot13, Lgi4, Tmem88, Syt15, Ddc, Cldn19, Mfsd12, Pdlim2, Sapcd2, Hr, Calml3, Foxp2, Ccdc146, Slc7a4, Kantr, Idi1, Slc1a3, Papln, Slc12a5, Gprc5c, Kif20b, Vsir, AABR07059875.2, Mgat3, Arhgef10l, Dock11, Kif15, Parpbp, Itpka, Rad51b, Ercc6l, Apbb1,	1086

	Mef2c, Rn50_13_0828.1, Dpysl2, Aldh3b1, Aim1l, Grem2, AABR07065113.1, Aspm, RGD1310335, Gas1, LOC100361087, Ckap2l, Irf7, Fzd2, Il33, Nfatc4, Rn50_14_0459.2, Zfp467, AABR07041794.1, Fndc1, Ddb2, Gpsm2, Plscr4, Rab11fip1, Ska1, Meis2, Gja4, Stard4, Gstm4, Cenpe, Hmcn1, Nme3, Ttc30b, RGD1309621, Fam189b, Aldh1a2, Rn60_20_0051.7, Cdh2, Acat2, Fam102a, Fah, AABR07049578.1, Camk1, Tlr5, Fam20a, Fam179a, Adam15, Mcam, Trerf1, Fam115c, Des, Flrt1, Aspn, Cdon, Pola2, Ptgdr2, Serpinb8, Podxl2, E2f7, Esrrg, Kif22, Plekha6, Ccnf, Rapgefl1, Suox, Hoxa11, Ly6l, Ablim1, Tead4, Kif20a, Ttc22, Hlx, Olfml2a, Wdr63, Ksr1, Stac2, Aifm2, Ctc1, Cacna1g, Cpt1c, AABR07055878.1, Atp8b3, Fam83d, Fam83e, Gatsl3, Aurkb, Id2, Scn1b, Klhl34, Ak1, Cyb561, Zfyve21, Elmo3, Plxnb1, Heg1, LOC680875, Bbox1, Akap12, Fam25a, LOC103694863, Rnf112, Pitpnm3, Zfhx2, Adamtsl4, Slc26a6, Pdlim3, Srebf2, Spag5, Mxi1, C1qtnf1, Fbxo32, Akap5, Pbx1, Serpinb1a, Vill, Ccr10, Mcee, AABR07001910.1, AABR07045487.1, Dbp, Trpm5, Pcdhgb8, Cmklr1, RGD1359290, Tns4, Lpl, Inpp5j, Spc24, Tgfbi, Celsr2, Tgm1, LOC102547216, Senp7, Il22ra1, Ddah2, LOC100911486, Map3k5, Ntf4, Kank2, Upk1b, AABR07021972.1, Tm4sf1, Espl1, B3galt4, Angptl2, Sgsm2, Enkur, Smarca1, Prkcg, Usp2, Hoxa10, Arhgef26, Cd59, Slc10a4, Tnnc1, Fdft1, Wdr6, Abcd1, Fmo4, Nrbp2, St3gal5, Gpr4, Hspb1, Rasa4, Sh3tc1, Exph5, Pkhd1l1, Atoh8, Gen1, Tecta, Synm, Asap2, AC130232.1, Ns5atp9, Slc44a2, Aass, Nkd1, Gjc2, Acadm, Gpd1l, Gas7, Ncaph, Arntl2, Slc4a9, Myh10, H2afy2, Fam92b, Mdga1, Pcdhb20, Hmgcs2, AC130862.3, Zbtb7c, Ermp1, Mertk, Sh3bp1, Syt11, Enpep, Coro6, Arhgap19, Tcea3, Vwde, Samd11, Aqp1, Oplah, Tpbgl, Adamtsl2, Pdcd4, Spc25, Mboat2, Tacc3, RGD1565693, Fos, Syt7, LOC103692128, Acpp, Synpo, Pttg1, Celsr1, Zadh2, St3gal2, Fsd1l, Plppr3, Scrn2, Calml4, Hsd17b4, Troap, Trim36, Spry1, Gpi, Decr1, Nhsl2, Sh3rf2, Col8a2, Mtus2, Sntg2, LOC100910979, Ube2c, Fxyd3, Pcdhb4, AABR07043395.1, Tmem37, Acaa2, Pfkm, Padi1, Ctnnal1, Ccdc138, Sept1, Xkr5, Palm, AABR07030520.1, Slfn5, Trim6, SNORA17, Fam69a, Ptprb, LOC103689925, Calcoco1, Cilp, Mob3b, Rhobtb3, Large2, Abhd15, Neurl1b, Wnt11, Rfx2, Matn3, Rn50_X_0691.2, Arhgef9, Rhbdl3, Smad6, Zc4h2, Phf24, Tst, Dixdc1, Mettl7a, Cd7, Nudt14, Fam198b, Duox1, Notch1, Wipf3, Speg, Gca, Rrm1, Foxo6, Crb2, Acsl3, Kcnd1, Smc4, Hpcal1, Fads1, Vegfd, Prkcdbp, Krt19, Srgap3, Nusap1, Chek2, Tmem151a, Prkch, Ccdc74a, Cnih2, Fras1, Mybpc2, Rrad, Neil3, Adam33, Bphl, Thra, Gnaz, Gja5, Duox2, Itgb8, Tbx18, Psd3, Itgb6, Cdc20, Slc12a6, Dhcr24, Gata6, Cxcl17, Gatm, Oip5, Myo7a, Dhrs3, Klhl24, Arhgef39, Cenpi, Mepe, RGD1563349, Elf5, Tle2, Fat2, Cdkn2c,	

	Sptbn1, Slc13a5, Tpx2, Wdr19, Rragd, Fgf18, Cdca3, S1pr5, Kif6, Sh2d4a, Lsp1, Sema6c, Smarca2, Ndc80, Lrrtm3, Chst4, LOC691170, Adamts3, Cobl, Adhfe1, Tnfaip8l3, Alkbh7, Tpra1, Dnali1, Kcnj9, RGD1306556, Nexn, Adcy5, Ckb, Ackr3, Prph, Islr, Tmem218, Gstm7, Sh3gl2, Sema6b, Syt2, Evpl, NEWGENE_2813, Unc5cl, Dsp, Evc, Chst12, Cd81, Hsf2bp, Gmip, Magi1, Xdh, Plpp2, Sox12, Tob1, Pcdhb19, Cacna2d2, Sh3bgrl2, Pde5a, Myh14, Gas6, Dock5, Klhl30, Ophn1, Acsm5, Hyal2, Ednra, Fhod3, Hoxa9, Nbeal2, Sned1, Adamtsl1, Cdk18, Dnah7, Skap2, AABR07043702.1, Sema3d, Fgd3, Slc29a2, AABR07006657.1, Pdk4, Btc, Pink1, Asb12, Gpr157, AABR07064878.1, Ankrd34a, Rab17, Klhdc8b, Exd1, Llgl2, Tet1, Ston1, Mfn2, Greb1, Cep70, Epb41l3, Frmd4b, Col12a1, Atp6v1g2, Abca1, Cyb5r3, LOC292543, Chrnb4, Frmpd4, Nr0b2, Dhcr7, Epb42, Cep162, Fry, Rbfox3, Krt13, Pcyt2, Erich4, LOC103690023, Slc24a3, AC141169.2, Hspb7, Mfap5, P4ha3, Ccdc82, Arvcf, Rom1, Prdm11, Pik3ip1, Gm2a, F11r, Arrb1, Cxcl13, Cln6, LOC103689947, Mrvi1, Tagln, Parm1, Dlgap5, Krt75, Zfp69, Sort1, Slc2a12, Upk3bl, Cct6b, Rab3a, Eef2k, Nmnat2, RGD1306227, Akr1c19, Tmem63c, Tinagl1, Fam64a, Fgf1, Wscd2, Dact2, Matn4, Pdgfb, Prrt2, Gldc, Mturn, Myh6, Itga9, Pald1, Smad9, Wdr76, Cyp2ab1, LOC691083, Clic3, Lgr4, Fam13a, Cfap52, Nbeal1, Igfbp3, Ccdc177, Rbm20, Wt1, Ltbp3, Gcdh, Map2k6, Rnf144b, Samhd1, Ddr1, Pla2g3, AABR07049695.2, Phyh, AABR07044036.1, Aunip, Sorbs1, Npepo, Ribc2, Ctxn1, Flot1, Antxr1, Slco2a1, Cxxc5, Gdf6, Pcyox1, AC134759.1, Anxa6, Slc28a2, Lipe, Ppp1r3c, Ccne1, Paqr8, Sult2b1, Aim1, Mapre2, Iqsec2, Daam2, Slc9a3r2, Elovl7, Inhbb, LOC679894, Sema4g, Tek, Trim63, Anxa8, Gmpr, AABR07049960.1, Htra3, Tm4sf4, Ldlr, Prkra, Sipa1, Slc16a4, Esyt3, RGD1309362, Rph3al, LOC102552640, Ppp1r13b, Hapln1, Prc1, -, Cdc42ep2, Idh1, LOC501406, Nkain4, Lfng, Scnn1a, Garnl3, Idnk, Lims2, Csdc2, Iqgap3, Tmem53, LOC103694864, Shroom3, Phactr1, Eci1, Mpp7, Hip1, Sord, Dmd, Tnfrsf25, Cpt2, Ngfr, Parvb, LOC102550543, Crip2, E2f8, LOC102555672, Slc22a18, Casq2, Fam117a, Kifc1, Ect2, Clmp, Atp6v0d2, Flot2, Synpo2, Tspan15, Afap1l2, Il17re, Cdhr2, Ppl, Cpm, AABR07040840.1, Gas2l3, Gpr39, Hoxd4, Atraid, Tbc1d2, Zfp579, Ntn4, A930018M24Rik, Sspn, Slc30a2, Foxn3, LOC108348320, Mknk2, Ndrg4, Tmem204, Tfdp2, Mastl, Paqr6, Lix1, Fkbp1b, Mme, Sh2d1a, Plcd1, AABR07024542.1, Tmem51, Plk1, Cryz, Wnt2b, Crnn, Fam180a, Rimbp2, Klhl31, Fam189a2, Bub1b, Pi15, Igsf8, Vwa1, AABR07058914.1, Gde1, Bhmt, AABR07030603.1, Hoxa6, Cyp39a1, AABR07065139.1, Spata13, Rab3d, Pik3r2, Tdp2, Skida1, Pcyox1l, Tsks, Abhd18, RGD1564899, Tmem9, Fhl1, Hadh,	

	Gatsl2, Zfp566, Scd2, Rab7b, AABR07048280.1, Ptpru, Dync1i1, Il17rd, AABR07030494.1, Fam13c, Ccnb1, Tm4sf20, LOC363337, Slc48a1, Slc9a3r1, Smim1, Kifc2, AC131360.1, Tyk2, Slc47a1, Ralgds, Casz1, AABR07000658.1, Fgfr3, E2f1, Inpp5k, Cldn9, Pdgfrb, Ggn, LOC100910915, Zkscan1, Hpse2, Sesn1, Ppm1k, Slc27a1, Zfpm1, Sec22c, Dab2, Snta1, Mgst3, Fyb, Lingo1, Ift80, Cldn15, Sv2a, Cdca2, Adgrb2, Ppfibp2, Acad10, Smim22, Asf1b, Sybu, -, Pcmtd2, E2f2, Akr1c12, Kif4a, RGD1564149, Cenpf, Dhdds, Gpr146, Scx, Fads2, Mycn, Itgbl1, Aif1l, B4galt5, Tmem179b, Comp, Rhpn2, Catip, Adrb1, Spsb1, Kif2c, Rassf7, Pnkd, AABR07034669.2, Ska3, Ehd2, Ndrg2, Mki67, Wisp2, Ypel1, Ccna2, Cyp2c11, Nynrin, Car11, LOC108348137, Insig1, Glul, Bnc2, Lypd1, Ccdc106, Ypel2, Sgcd, Padi3, Herc3, Zfyve28, Col4a4, Il1rn, Bcam, Melk, Rnf207, Inpp5f, Rspo3, Sema3a, Cracr2b, Smtnl2, Popdc2, Ahsp, Ptn, A3galt2, Dlg3, Masp1, Hacd4, Ptpn13, Aldoc, LOC685849, Adgrg1, Pcyt1b, Cdc14a, SNORA71, Top2a, Esco2, Racgap1, Fam131a, B3gnt8, Glrx, Pdgfra, Ankle1, Csrp2, Slc38a1, Mpzl2, Socs1, Adamts1, Wwtr1, Tagap, Pdk2, Prr15l, LOC679342, Papss1, Kcnn4, Pcdha4, Rundc3a, LOC103694874, Ca5b, March2, Slc12a8, Mxra8, Tmem38a, Tcp11l2, Dedd2, Hif3a, Rflnb, Ncapg, Fam210b, Fcho1, Agap2, Armc2, Aldh5a1, Krt15, Upk3b, Nuf2, Kif14, Colec12, Mbd4, RT1-N2, Mtmr3, Impa2, Dennd2d, Ccdc160, Aox1, Ip6k3, RT1-T24-3, Kcnh2, Mns1, Mif4gd, Gucy1a3, NEWGENE_1308171, Rn50_11_0160.1, St6galnac6, Etfbkmt, Pot1b, Tns1, Iqgap2, Fnbp1, Tgm7l1, Tspoap1, Mis18bp1, Gnptab, Cgnl1, Zfp606, Lrrc56, Bmf, Klhl33, Efna2, Shcbp1, Tceal5, Slc44a4, Anlnl1, Nrxn2, Cep72, Pcdha2, Rn60_10_0924.2, AABR07041411.1, Cit, Tgfb3, Ctdspl, Map4k1, Ms4a8, Sdpr, Camk2n1, Scara5, Apcdd1, Ptprf, Lmcd1, Sema6a, Gfap, Rgs22, Mpp2, Atcay, LOC100364027, Adamts9, Cspg4, Sgo1, Tbc1d8, Socs3, Selenop, Abca2, Nipal3, Fzd7, LOC102553180, Hspb6, Kcnip3, Susd2, Ptgs1, Arhgef19, Myl9, Arid4b, Anpep, Gsta4, Kif11, Sytl1, Dusp14, AC121639.1, Ntn3, Xirp1, Dzip3, LOC100911668, Amer1, Ctla2a, Ulk4, Ccng2, Tmcc3, Dysf, Syne2, Ttyh3, Car3, Akap7, Itga1, Stk26, Adamtsl3, Cdc42bpg, LOC498222, Cenpt, AABR07001923.1, Sgo2, Rwdd2a, S100a4, Nrm, Car1, Nbl1, LOC500300, AABR07049695.3, Lmod3, Mxd4, Cd36, Pdpn, Tmem14a, Bin2, Slc2a10, Lrrn4, Lztfl1, RGD1309534, Wbscr28, Apold1, Nol4l, Capn5, Tk1, Pacsin3, Ppp1r1b, LOC100912611, Tekt1, Depdc1b, Vtcn1, Mmp24, Mmp11, Syne3, Ech1, Dact3, Cenpw, Slc29a1, Gli2, Omd, Lpin1, Otogl, Slc8a1, Kazald1, Arhgap33, C1qtnf6, Medag, Kcnmb4, RGD1559896, AABR07017208.1, Prkcz, Htr1b, Ndrg1, Rapgef3, Irf6, Car9, Abtb1,	

	Foxd2, Utrn, Cdt1, Rnf145, Arhgap18, LOC691143, Tshz1, Hist1h1b, Nrp1, Slc39a10, Arhgef10, Igf2bp1, Rgma, Coq8a, Knstrn	

Table. S2：Lists of genes affected by α-MG group

Transcriptome results	Genes	Total number
Down-regulated genes	Creb5, Entpd1, Nppb, Csf3, Vegfa, Acsm1, Kbtbd8, Nhs, Fgf21, Fam81b, Faslg, Rgs9, Slc41a2, Plcl1, Csf2, SNORA73, SNORD93, Nox1, Etv4, AABR07015674.1, LOC688459, Atp8b4, Pycr1, Klra2, Cyp4f39, Tgfa, Pld5, Slc25a37, Nlrp3, Il23a, Qtrt2, Il9r, Rap1gap2, Dnajc25, Piezo2, St6galnac1, Steap1, Gpr155, Cdr2, Jaml, Oit3, Abcc8, Abca14, Cpeb2, Ifi27l2b, LOC103691744, Ppm1h, Cacna1d, AABR07067469.1, Sele, Cd300lf, Bcat1, Odc1, Serpinb2, Pak1ip1, SNORA32, Col4a3, Cd44, Slc7a5, Clec10a, Plekhs1, Tslp, Abcg1, Plet1, Srm, Pcdh12, Map1b, Tmem154, LOC100911453, Adam23, Pramef27, AABR07058805.1, Gar1, Ttc39b, Cyp19a1, AABR07047844.1, Cacna1h, AABR07014239.1, Akna, Ercc1, Tnfaip6, Il24, Tnfsf18, Klf6, Zeb2, Cox6a2, Acot2, Slco5a1, Ptgs2, Il1rl1, Fyn, Serp1, Vldlr, Etnppl, LOC100911440, Dll4, Vat1l, Etv5, Cd69, Clca2, LOC102551435, AC128848.1, Adm2, Gramd1b, Ubash3b, Gadd45a, Cd160, Mmp13, Elavl2, Nlrx1, Fam185a, Ifrd1, Kctd19, Has2, Fosl1, Tmem158, Hs3st5, Fhl2, AABR07066820.1, LOC102546376, Cd28, Trib3, Card11, Noct, Aox2, Ciart, Hspa4l, Grhl1, Cyp26b1, Shc4, Slc7a11, Kif21b, Ogfod1, Cenpn, Plcd4, Prss22, Sytl2, AABR07050530.1, Ddr2, Scube2, Lif, LOC103692719, Gpatch4, LOC500877, Slc16a14, Atf6, AABR07017902.1, Trpc6, Nnmt, Dapk1, AABR07054460.4	151
Up-regulated genes	Tmem140, Gpr37l1, Kank4, LOC100360908, Nod1, Mmd, Mybphl, Csgalnact1, Ptk2b, Map1a, Mboat1, Adamts7, Krt14, Lss, Ovol1, Hrct1, Nxph3, Irf9, Lgi4, Syt15, Hr, Mgat3, Apbb1, Dpysl2, Grem2, Gas1, Serpinb1a, Dbp, Trpm5, Tns4, Celsr2, Ddah2, LOC100911486, LOC100361087, Irf7, Il33, Zfp467, AABR07041794.1, Plscr4, Ttc30b, Fam189b, Aldh1a2, Fam102a, Fah, Arg1, Fam179a, Mcam, Fam115c, Pola2, Ldhb, Ly6l, Ablim1, Ttc22, Olfml2a, Cacna1g, Atp8b3, Fam83d, Gatsl3, Id2, Ak1, LOC103694863, Pitpnm3, Pdlim3, C1qtnf1, Krt17, Upk1b, B3galt4, Angptl2, Rtp4, Usp2, Tnnc1, Fdft1, Abcd1, St3gal5, Rasa4, Sh3tc1, Atoh8, Gjc2, Gas7, Arntl2, Fam92b, Zbtb7c, Coro6, Tcea3, Synpo, Plppr3, Calml4, Sh3rf2, Col8a2, LOC100910979, Pfkm, Ctnnal1, Trim6, Large2, Matn3, Rhbdl3, Zc4h2, Phf24, Fam198b, Ckmt1, Kcnd1, Fads1, Krt19, Srgap3, Nusap1, Chek2, Ccdc74a, Cnih2, Fras1, Mybpc2, Thra, Gnaz, Duox2, Cdc20, Dhcr24, Cxcl17, Oip5, Dhrs3, Rragd, Fgf18, Cdca3, S1pr5, Sh2d4a, Lsp1, Sema6c, Chst4, Cobl, Adhfe1, RGD1306556, Ckb,	324

	Ackr3, Prph, Islr, Evpl, Chst12, Pcdhb19, Myh14, Gas6, Klhl30, Pidd1, Sned1, Fgd3, Pdk4, Btc, Asb12, Ankrd34a, Elf3, Llgl2, Mfn2, RT1-S3, Frmd4b, LOC292543, Dhcr7, P4ha3, Pik3ip1, Arrb1, Cln6, LOC103689947, Tagln, Parm1, Krt75, Fam84b, Nmnat2, Tmem63c, Tinagl1, Dact2, Prrt2, Rimkla, LOC691083, Cfap52, Ccdc177, Rnf144b, Samhd1, Phyh, Npepo, Ctxn1, Antxr1, AC134759.1, Anxa6, Lipe, Paqr8, Slc9a3r2, Anxa8, Plxnb3, Htra3, Ldlr, Sipa1, RGD1309362, Cdc42ep2, Ifi47, Idh1, Lfng, Idnk, Csdc2, Iqgap3, Phactr1, Eci1, Mpp7, Tnfrsf25, Ngfr, Parvb, Duoxa2, Apol3, Crip2, Clmp, Flot2, Afap1l2, Ppl, Atraid, Tbc1d2, Isg15, Mknk2, Tfdp2, Mme, Fam180a, Vwa1, Rspo1, AABR07065139.1, Spata13, Gatsl2, AABR07048280.1, Ptpru, Cgn, Il17rd, AABR07030494.1, Slc48a1, Slc9a3r1, Smim1, AABR07000658.1, Fgfr3, Sesn1, Gpc3, Snta1, Cldn15, Adgrb2, Ppfibp2, Gpr146, Scx, Fads2, Mycn, Aif1l, B4galt5, Rhpn2, Spsb1, Kif2c, Rassf7, Wisp2, Ypel1, Nynrin, Insig1, Glul, Ypel2, Il1rn, Rnf207, Sema3a, Smtnl2, Popdc2, Ahsp, A3galt2, Fmod, LOC685849, Adgrg1, Pcyt1b, B3gnt8, Pdgfra, Csrp2, Slc38a1, Mpzl2, Pdk2, Prr15l, Ca5b, Mxra8, Tcp11l2, Hif3a, Rflnb, Wnt10a, Fam210b, Krt15, Col14a1, Upk3b, Colec12, Ip6k3, Kcnh2, Rn50_11_0160.1, Etfbkmt, Tns1, Lrrc56, Nrxn2, Cit, Tgfb3, Ctdspl, Sdpr, Scara5, Mpp2, Adamts9, Cspg4, Tbc1d8, Kcnip3, Myl9, Elk1, Xirp1, Fbp2, Ccng2, Akap7, Cdc42bpg, Rwdd2a, Nbl1, LOC500300, Mxd4, Pdpn, Lrrn4, RGD1309534, Ppp1r1b, Vtcn1, Mmp11, Dact3, Omd, Slc8a1, Medag, Rapgef3, Irf6, Rgma, Coq8a, Knstrn	

Table. S3: Lists of genes affected by LPS and α-MG group

Transcriptome results	Genes	Total number
Control and Mangostin down-regulate genes with the same trend	Creb5, Entpd1, Nppb, Csf3, Vegfa, Acsm1, Kbtbd8, Nhs, Fgf21, Faslg, Rgs9, Slc41a2, Plcl1, Csf2, SNORA73, SNORD93, Nox1, Etv4, AABR07015674.1, LOC688459, Atp8b4, Pycr1, Klra2, Cyp4f39, Tgfa, Pld5, Slc25a37, Nlrp3, Il23a, Qtrt2, Il9r, Rap1gap2, Dnajc25, Piezo2, St6galnac1, Steap1, Gpr155, Cdr2, Jaml, Oit3, Abcc8, Abca14, Cpeb2, Ifi27l2b, LOC103691744, Ppm1h, Cacna1d, AABR07067469.1, Sele, Cd300lf, Bcat1, Odc1, Serpinb2, Pak1ip1, SNORA32, Col4a3, Cd44, Slc7a5, Clec10a, Plekhs1, Tslp, Abcg1, Plet1, Srm, Pcdh12, Map1b, Tmem154, LOC100911453, Adam23, Pramef27, AABR07058805.1, Gar1, Ttc39b, Cyp19a1, AABR07047844.1, Cacna1h, AABR07014239.1, Akna, Ercc1, Tnfaip6, Il24, Tnfsf18, Klf6, Zeb2, Cox6a2, Acot2, Slco5a1, Ptgs2, Il1rl1, Fyn, Serp1, Vldlr,	149

	Etnppl, LOC100911440, Dll4, Vat1l, Etv5, Cd69, Clca2, LOC102551435, AC128848.1, Adm2, Gramd1b, Ubash3b, Gadd45a, Cd160, Mmp13, Elavl2, Nlrx1, Fam185a, Ifrd1, Kctd19, Has2, Fosl1, Tmem158, Hs3st5, Fhl2, AABR07066820.1, LOC102546376, Cd28, Trib3, Card11, Noct, Aox2, Ciart, Hspa4l, Grhl1, Cyp26b1, Shc4, Slc7a11, Kif21b, Ogfod1, Cenpn, Plcd4, Prss22, Sytl2, AABR07050530.1, Ddr2, Lif, LOC103692719, Gpatch4, LOC500877, Slc16a14, Atf6, AABR07017902.1, Trpc6, Nnmt, Dapk1, AABR07054460.4	
Control and Mangostin up-regulate genes with the same trend	Tmem140, Kank4, LOC100360908, Nod1, Mmd, Mybphl, Csgalnact1, Ptk2b, Map1a, Mboat1, Lss, Ovol1, Hrct1, Nxph3, Irf9, Lgi4, Syt15, Hr, Mgat3, Apbb1, Dpysl2, Grem2, Gas1, Serpinb1a, Dbp, Trpm5, Tns4, Celsr2, Ddah2, LOC100911486, LOC100361087, Irf7, Il33, Zfp467, AABR07041794.1, Plscr4, Ttc30b, Fam189b, Aldh1a2, Fam102a, Fah, Fam179a, Mcam, Fam115c, Pola2, Ly6l, Ablim1, Ttc22, Olfml2a, Cacna1g, Atp8b3, Fam83d, Gatsl3, Id2, Ak1, LOC103694863, Pitpnm3, Pdlim3, C1qtnf1, Upk1b, B3galt4, Angptl2, Usp2, Tnnc1, Fdft1, Abcd1, St3gal5, Rasa4, Sh3tc1, Atoh8, Gjc2, Gas7, Arntl2, Fam92b, Zbtb7c, Coro6, Tcea3, Synpo, Plppr3, Calml4, Sh3rf2, Col8a2, LOC100910979, Pfkm, Ctnnal1, Trim6, Large2, Matn3, Rhbdl3, Zc4h2, Phf24, Fam198b, Kcnd1, Fads1, Krt19, Srgap3, Nusap1, Chek2, Ccdc74a, Cnih2, Fras1, Mybpc2, Thra, Gnaz, Duox2, Cdc20, Dhcr24, Cxcl17, Oip5, Dhrs3, Rragd, Fgf18, Cdca3, S1pr5, Sh2d4a, Lsp1, Sema6c, Chst4, Cobl, Adhfe1, RGD1306556, Ckb, Ackr3, Prph, Islr, Evpl, Chst12, Pcdhb19, Myh14, Gas6, Klhl30, Sned1, Fgd3, Pdk4, Btc, Asb12, Ankrd34a, Llgl2, Mfn2, Frmd4b, LOC292543, Dhcr7, P4ha3, Pik3ip1, Arrb1, Cln6, LOC103689947, Tagln, Parm1, Krt75, Nmnat2, Tmem63c, Tinagl1, Dact2, Prrt2, LOC691083, Cfap52, Ccdc177, Rnf144b, Samhd1, Phyh, Npepo, Ctxn1, Antxr1, AC134759.1, Anxa6, Lipe, Paqr8, Slc9a3r2, Anxa8, Htra3, Ldlr, Sipa1, RGD1309362, Cdc42ep2, Idh1, Lfng, Idnk, Csdc2, Iqgap3, Phactr1, Eci1, Mpp7, Tnfrsf25, Ngfr, Parvb, Crip2, Clmp, Flot2, Afap1l2, Ppl, Atraid, Tbc1d2, Mknk2, Tfdp2, Mme, Fam180a, Vwa1, AABR07065139.1, Spata13, Gatsl2, AABR07048280.1, Ptpru, Il17rd, AABR07030494.1, Slc48a1, Slc9a3r1, Smim1, AABR07000658.1, Fgfr3, Sesn1, Snta1, Cldn15, Adgrb2, Ppfibp2, Gpr146, Scx, Fads2, Mycn, Aif1l, B4galt5, Rhpn2, Spsb1, Kif2c, Rassf7, Wisp2, Ypel1, Nynrin, Insig1, Glul, Ypel2, Il1rn, Rnf207, Sema3a, Smtnl2, Popdc2, Ahsp, A3galt2, LOC685849, Adgrg1, Pcyt1b, B3gnt8, Pdgfra,	298

	Csrp2, Slc38a1, Mpzl2, Pdk2, Prr15l, Ca5b, Mxra8, Tcp11l2, Hif3a, Rflnb, Fam210b, Krt15, Upk3b, Colec12, Ip6k3, Kcnh2, Rn50_11_0160.1, Etfbkmt, Tns1, Lrrc56, Nrxn2, Cit, Tgfb3, Ctdspl, Sdpr, Scara5, Mpp2, Adamts9, Cspg4, Tbc1d8, Kcnip3, Myl9, Xirp1, Ccng2, Akap7, Cdc42bpg, Rwdd2a, Nbl1, LOC500300, Mxd4, Pdpn, Lrrn4, RGD1309534, Ppp1r1b, Vtcn1, Mmp11, Dact3, Omd, Slc8a1, Medag, Rapgef3, Irf6, Rgma, Coq8a, Knstrn	

Table. S4: Difference GO list for LPS group (14)

GO ID	GO name	Category	Genes	Total number
GO:0006954	inflammatory response	BP	Cxcl1, Cxcl2, Cxcl3, Tspan2, Jak2, Nfkb2, Tnfrsf9, Relb, Cxcl10, Cxcl13, Relt, Epha2, Tnfaip3, Prkcz, Csf1, Il1rn, Cyp26b1, Bdkrb1, Ptgs1, Ptgs2, Nfkbiz, Elf3, Il23a, Il17re, Olr1, Ccl20, Nod1, Nod2, Ccl2, Ccl5, Ccl7, Cspg4, Tnfrsf25, Tnfrsf26, Zc3h12a, Ngfr, C3, Cd40, Tnf, Gbp5, Il1a, Nlrp3, Nlrp6, Afap1l2, Cx3cl1, Il17f, Nlrx1, Chst4, Sele, Selp, Pparg, Il6, Casp4, Tlr2, Tlr5, Kdm6b, Ripk2, Fas, Acod1, Itgb6, S100a7a, Bmp6, Ffar2	63
GO:0042981	regulation of apoptotic process	BP	Tgfb3, Chek2, Jak2, Tnfrsf9, Fyn, Relt, Arrb1, Rassf6, Nme3, Dedd2, Map3k8, Lefty1, Tnfaip8l3, Egr1, Inhbb, Inhbe, Nod1, Nod2, Myc, Bnc2, Tnfrsf25, Tnfrsf26, Bid, Ngfr, Cd40, Map2k6, Card11, Hip1, Traf1, Traf2, Traf3, Casp12, Bcl3, Gdf6, Birc3, Map4k1, Ank2, Il6, Pmaip1, Casp4, Stk26, Pink1, Ripk2, Fas, Cflar, Bmp6	46
GO:0032496	response to lipopolysaccharide	BP	Cxcl1, Cxcl2, Cxcl3, Jak2, Prdx3, Tnfrsf9, Cxcl10, Cxcl13, Relt, Slpi, Cebpb, Bdkrb1, Ptgs2, Nfkbia, Noct, Nod2, Tnfrsf25, Tnfrsf26, Ngfr, Cd40, Rpl13a, Tnf, Trim6, Ednra Cyp27b1, Fas, Acod1	27
GO:0045429	positive regulation of nitric oxide biosynthetic process	BP	Jak2, Ptk2b, Ptgs2, Icam1, Klf4, Cd36, Tnf, Ddah2, Il6, Tlr2, Tlr5, Sod2	12
GO:0032755	positive regulation of interleukin-6 production	BP	Vegfd, Tslp, Rab7b, Nod1, Nod2, Cd36, Tnf, Il1a, Il33, Adora2b, Il6, Tlr2, Ripk2	13
GO:0097190	apoptotic signaling pathway	BP	Anxa6, Tnfrsf9, Relt, Ifi27l2b, Tnfrsf25, Tnfrsf26, Ngfr, Cd28, Cd40, Tnf, Hip1, Uaca, Dapk1, Fas	14

GO:0019221	cytokine-mediated signaling pathway	BP	Ifngr2, Socs1, Socs2, Socs3, Lrrtm3, Jak2, Il1rn, Duox1, Duox2, Lrtm2, Il6st, Il17rd Il17re, Ccl2, Klf6, Flrt1, Flrt2, Grem2, Il1a, Cx3cl1, Il20rb, Irf5, Il6, Il22ra1, Aspn	25
GO:0050729	positive regulation of inflammatory response	BP	Jak2, Tslp, Nfkbia, Ldlr, Tnfsf18, Il17ra, Pdcd4, Il1rl1, Tnf, Il33, Ednra, Tlr2, Hyal2	13
GO:0043122	regulation of I-kappaB kinase/NF-kappaB signaling	BP	Nod1, Nod2, Angpt1, Tnf, Nlrp3, Nlrp6, Nlrx1, Hspb1, Optn	9
GO:0071345	cellular response to cytokine stimulus	BP	Socs1, Csf3, Foxh1, Nfkbia, Mme, Il17ra, Lef1	7
GO:0002682	regulation of immune system process	BP	Siae, Ggt1	2
GO:0006925	inflammatory cell apoptotic process	BP	Fas	1
GO:0006979	response to oxidative stress	BP	Jak2, Prdx3, Ppp1r15b, Idh1, Hmox1, Car3, Ptgs1, Ptgs2, Ercc1, Ercc8, Mmp9, Dgkk, Upk3bl, Pink1, Sod2	15
GO:0050725	positive regulation of interleukin-1 beta biosynthetic process	BP	Egr1	1

Table. S5: Difference GO list for α-MG group (15)

GO ID	GO name	Category	Genes	Total number
GO:0010942	positive regulation of cell death	BP	Ptgs2, Nod1, Mycn	3
GO:0001558	regulation of cell growth	BP	Wisp2, Cd44, Htra3	3
GO:0001819	positive regulation of cytokine production	BP	Card11, Il33	2
GO:0001666	response to hypoxia	BP	Acot2, Tgfb3, Vegfa, Hif3a, Slc8a1	5
GO:0032727	positive regulation of	BP	Irf7	1

	interferon-alpha production			
GO:0016064	immunoglobulin mediated immune response	BP	Irf7	1
GO:0033962	cytoplasmic mRNA processing body assembly	BP	Noct	1
GO:0032693	negative regulation of interleukin-10 production	BP	Il23a	1
GO:0071345	cellular response to cytokine stimulus	BP	Csf3, Mme	2
GO:0032755	positive regulation of interleukin-6 production	BP	Tslp, Nod1, Il33	3
GO:0035772	interleukin-13-mediated signaling pathway	BP	Cd300lf	1
GO:0070970	interleukin-2 secretion	BP	Card11	1
GO:0045519	interleukin-23 receptor binding	BP	Il23a	1
GO:0005125	cytokine activity	BP	Tgfb3, Vegfa, Tslp, Csf2, Csf3, Il1rn, Il23a, Tnfsf18, Grem2, Il24, Lif, Il33	12
GO:0006915	apoptotic process	BP	Chek2, Tns4, Rassf7, Sema3a, Mfn2, Rnf144b, Hif3a, Nod1, Kcnip3, Ngfr, Trib3, Shc4, Lsp1, Mknk2, Il24, Pidd1, Fgfr3, Dapk1, Apbb1	19

Table. S6: Difference pathway list for LPS group (9)

KEGG name	Genes	Total number
TNF signaling pathway	Atf4, Bcl3, Birc3, Ccl2, Ccl20, Ccl5, Cebpb, Cflar, Creb3l1, Creb5, Csf1, Csf2, Cx3cl1, Cxcl1, Cxcl10, Cxcl2, Cxcl3, Fas, Fos, Icam1, Il6, Lif, Map2k6, Map3k5, Map3k8, Mapk11, Mmp9, Nfkbia, Nod2, Pgam5, Pik3r2, Ptgs2, Sele, Socs3, Tnf, Tnfaip3, Traf1, Traf2, Traf3, Vcam1	41
IL-17 signaling pathway	Ccl2, Ccl20, Ccl7, Cebpb, Csf2, Csf3, Cxcl1, Cxcl10, Cxcl2, Cxcl3, Fos, Fosl1, Ikbke, Il17f, Il17ra, Il17re, Il6, Lcn2, Mapk11, Mmp13, Mmp9, Nfkbia, Ptgs2, Tnf, Tnfaip3, Traf2, Traf3	27
MAPK signaling pathway	Angpt1, Arrb1, Atf4, Cacna1d, Cacna1g, Cacna1h, Cacna1i, Cacna1s, Cacna2d2, Csf1, Ddit3, Dusp4, Dusp8, Efna2, Epha2, Fas, Fgf1, Fgf18, Fgf21, Fgfr2, Fgfr3, Fos, Gadd45a, Hspb1, Il1a, Map2k6, Map3k5, Map3k6, Map3k8, Map4k1, Mapk11, Mef2c, Mknk1, Mknk2, Myc, Nfkb2, Ngfr, Pdgfb, Pdgfra, Pdgfrb, Prkcg, Relb,	51

	Rps6ka2, Srf, Tek, Tgfa, Tgfb3, Tnf, Traf2, Vegfa, Vegfd	
p53 signaling pathway	Bid, Ccnb1, Ccnd2, Ccne1, Ccng2, Chek2, Cycs, Ddb2, Fas, Gadd45a, Igfbp3, Pmaip1, Sesn1, Sesn2	14
Apoptosis	Atf4, Bid, Birc3, Casp12, Cflar, Cycs, Ddit3, Ern1, Fas, Fos, Gadd45a, Map3k5, Nfkbia, Pik3r2, Pmaip1, Ptpn13, Tnf, Traf1, Traf2	19
Jak-STAT signaling pathway	Aox1, Aox2, Ccnd2, Csf2, Csf3, Fhl1, Gfap, Ifngr2, Il20rb, Il22ra1, Il23a, Il24, Il6, Il6st, Il9r, Irf9, Jak2, Lif, Myc, Pdgfb, Pdgfra, Pdgfrb, Pik3r2, Socs1, Socs2, Socs3, Thpo, Tslp, Tyk2	29
Inflammatory bowel disease (IBD)	Ifngr2, Il17f, Il18rap, Il1a, Il23a, Il6, Nod2, Tgfb3, Tlr2, Tlr5, Tnf	11
NF-kappa B signaling pathway	Birc3, Card11, Cd40, Cflar, Cxcl2, Icam1, Ltb, Nfkb2, Nfkbia, Ptgs2, Relb, Tnf, Tnfaip3, Traf1, Traf2, Traf3, Vcam1	17
Cytokine-cytokine receptor Interaction	Ackr3, Ccl2, Ccl20, Ccl5, Ccl7, Ccr10, Cd40, Csf1, Csf2, Csf3, Cx3cl1, Cxcl1, Cxcl10, Cxcl11, Cxcl13, Cxcl16, Cxcl2, Cxcl3, Fas, Ifngr2, Il17f, Il17ra, Il17re, Il18rap, Il1a, Il20rb, Il22ra1, Il23a, Il24, Il6, Il6st, Il9r, Inhbb, Inhbe, Lif, Ltb, Ngfr, Pdgfb, Pdgfra, Pdgfrb, Relt, Tgfb3, Thpo, Tnf, Tnfrsf25, Tnfrsf9, Tnfsf13, Tnfsf15, Tnfsf18, Tnfsf8, Tslp, Vegfa, Vegfd	53

Table. S7: Difference pathway list for α-MG group (5)

KEGG name	Genes	Total number
Cytokine-cytokine receptor interaction	Ackr3, Csf2, Csf3, Il23a, Il24, Il9r, Lif, Ngfr, Pdgfra, Tgfb3, Tnfrsf25, Tnfsf18, Tslp, Vegfa	14
Jak-STAT signaling pathway	Aox2, Csf2, Csf3, Il23a, Il24, Il9r, Irf9, Lif, Pdgfra, Tslp	10
MAPK signaling pathway	Arrb1, Cacna1d, Cacna1g, Cacna1h, Elk1, Fgf18, Fgf21, Fgfr3, Gadd45a, Mknk2, Ngfr, Pdgfra, Tgfa, Tgfb3, Vegfa	15
p53 signaling pathway	Ccng2, Chek2, Gadd45a, Pidd1, Sesn1	5
TNF signaling pathway	Creb5, Csf2, Ifi47, Lif, Ptgs2, Sele	6
